# Metabolic host responses to infection by intracellular bacterial pathogens

**DOI:** 10.3389/fcimb.2013.00024

**Published:** 2013-07-09

**Authors:** Wolfgang Eisenreich, Jürgen Heesemann, Thomas Rudel, Werner Goebel

**Affiliations:** ^1^Lehrstuhl für Biochemie, Center of Isotopologue Profiling, Technische Universität MünchenGarching, Germany; ^2^Max von Pettenkofer-Institut für Hygiene und Medizinische Mikrobiologie, Ludwig-Maximilians-Universität MünchenMünchen, Germany; ^3^Lehrstuhl für Mikrobiologie, Biozentrum Universität WürzburgWürzburg, Germany

**Keywords:** metabolism of mammalian cells, regulation of metabolic pathways, cancer cells, intracellular bacteria, common (“core”) and specific metabolic host responses, virulence-associated factors, antibacterial therapy

## Abstract

The interaction of bacterial pathogens with mammalian hosts leads to a variety of physiological responses of the interacting partners aimed at an adaptation to the new situation. These responses include multiple metabolic changes in the affected host cells which are most obvious when the pathogen replicates within host cells as in case of intracellular bacterial pathogens. While the pathogen tries to deprive nutrients from the host cell, the host cell in return takes various metabolic countermeasures against the nutrient theft. During this conflicting interaction, the pathogen triggers metabolic host cell responses by means of common cell envelope components and specific virulence-associated factors. These host reactions generally promote replication of the pathogen. There is growing evidence that pathogen-specific factors may interfere in different ways with the complex regulatory network that controls the carbon and nitrogen metabolism of mammalian cells. The host cell defense answers include general metabolic reactions, like the generation of oxygen- and/or nitrogen-reactive species, and more specific measures aimed to prevent access to essential nutrients for the respective pathogen. Accurate results on metabolic host cell responses are often hampered by the use of cancer cell lines that already exhibit various de-regulated reactions in the primary carbon metabolism. Hence, there is an urgent need for cellular models that more closely reflect the *in vivo* infection conditions. The exact knowledge of the metabolic host cell responses may provide new interesting concepts for antibacterial therapies.

## Introduction

Interactions between eukaryotic organisms and prokaryotes are ubiquitous. These encounters may affect the metabolism of the interacting partners in different ways, resulting in beneficial, neutral, or detrimental outcome for the partners. Highly stable metabolic win-win adaptations are manifested in the endosymbiotic interactions taking place between many invertebrates (especially protists and insects) and different bacteria (reviewed by Chaston and Goodrich-Blair, 2010). Long-lasting beneficial (or at least neutral) metabolic adaptations also occur between mammalians and their different microbiota, e.g., the microbiota of the gastrointestinal tract. However, these latter beneficial adaptations are less stable than the former ones and are often disturbed by external factors, for example by infections with bacterial pathogens (Lupp et al., 2007; Stecher et al., 2007). Metabolic adaptations occurring in eukaryotic hosts upon acute infection by bacterial pathogens are ultimately always conflicting, as the host tries to eliminate the invading pathogen while the pathogen tries to profit from host nutrients and other metabolites to satisfy its bioenergetic and biosynthetic requirements thereby damaging the host. Frequent metabolic changes may therefore occur on the part of the pathogen and on the part of the host in the course of an infection. The elucidation of metabolic host responses to bacterial infections—the topic of this review—is of great importance for the understanding of bacterial pathogenesis. Our present knowledge on this aspect is, however, still rather fragmentary.

Many studies have been carried out in the past to elucidate the responses of different hosts to bacterial infections mainly by transcriptomic and proteomic approaches. The main focus of most of these investigations has been placed on the pathogen-triggered immune responses, inflammation processes, endosomal vesicle formation, and trafficking (especially for intracellular pathogens), host cell survival, apoptosis, and autophagy (for reviews, see for example: Manger and Relman, 2000; Jenner and Young, 2005; Tran Van Nhieu and Arbibe, 2009), while the impact of the bacterial infections on the metabolism of the affected hosts has been rather set aside.

Yet, most of the more extensively studied cell responses, in particular cell transformation, inflammation, and specific immune response, are linked to carbon, nitrogen, and/or energy metabolism by shared enzymes, signaling pathways, and/or transcriptional regulators (Hsu and Sabatini, 2008; Mathis and Shoelson, 2011; Andersen and Kornbluth, 2013; O'Neill and Hardie, 2013).

To a considerable extent, the missing in-depth knowledge of metabolic host responses to bacterial infections is due to the fact that the elucidation of the metabolic changes in the host and the bacterial pathogen during infections poses major experimental challenges in terms of the infection models and the analytical methods (discussed in some detail in the text below). Metabolism is a complex phenomenon that comprises multiple steps from gene to mRNA to the active enzyme. In general, message levels cannot be equated with enzyme protein levels nor can enzyme protein levels be equated with enzyme activity as documented in many instances. Metabolic flux is—as elaborated especially by Westerhoff and colleagues (e.g., van Eunen et al., 2011)—subject to “hierarchical regulation” involving changes in enzyme levels, and to “metabolic regulation” including modulations of the activity of pre-existing enzymes.

At present, analysis of metabolic host responses to bacterial pathogens is probably best accessible for the interactions between mammalian cells and intracellular bacterial pathogens, since the infected host cell represents a fairly well-defined metabolic entity.

**Figure 1:**
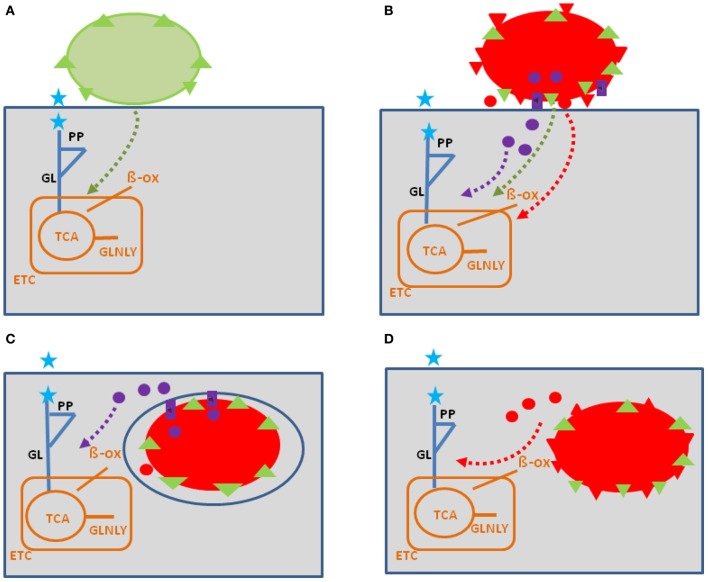
**Model showing possible interactions of common bacterial surface components and pathogen-specific virulence factors with the host cell metabolism in the cytosol (blue lines) and in the mitochondria (orange)**. The schematic view of the host cell (gray box) metabolism comprises glucose uptake (blue star), glycolysis (GL), pentose-phosphate shunt (PP), tricarboxylic acid cycle (TCA), glutaminolysis (GLNLY), β-oxidation of fatty acids (β-ox), and the electron transport chain (ETC) essential for aerobic respiration. **(A)** Interaction with non-pathogenic bacteria (green sphere) which may trigger common metabolic host cell responses (green dashed arrows; see text for definition) mainly via cell envelope structures (e.g., PG, LPS, LTA—green triangles). **(B)** Extracellular bacterial pathogens (red sphere) may trigger in addition to these common responses (green dashed arrows), specific metabolic host cell responses via cell-bound or secreted virulence factors (red triangles and red circles), as well as via specific secretion systems (e.g., T3SS or T4SS; purple boxes) translocated effector proteins (purple circles). **(C)** Intracellular bacterial pathogens replicating in membrane-surrounded vacuoles within the host cell could trigger metabolic host responses via translocated effector proteins (purple circles). **(D)** Intracellular bacterial pathogens replicating in the host cell's cytosol could trigger metabolic host responses via secreted or cell-bound virulence factors (red triangles and circles). For abbreviations and further details, see text.

Intracellular bacterial pathogens may in principle interfere with host metabolism by means of their common cell envelope structures [especially peptidoglycans (PG), lipopolysaccharides (LPS), lipoteichoic acids (LTA), respectively] and specific virulence-associated factors. This interference may occur on all levels, i.e., regulation and expression of nutrient sensors and transporters or regulators involved in the expression of key enzymes essential for catabolism, anabolism, and energy supply as well as in the modulation of activity of these enzymes (for a schematic overview, see Figure 1).

The mammalian hosts (mainly discussed here) possess a variety of differentiated cells with different overall metabolic activity and differently regulated metabolic pathways. The metabolic activity of these “normal” cells is often low but can be activated by various external stimuli, including microbial components (O'Neill and Hardie, 2013). This is in contrast to most cancer cells (often used as model host cells for the study of intracellular bacteria). In these cells some catabolic and anabolic pathways are always highly up-regulated, while aerobic respiration is strongly reduced even in the presence of oxygen, a metabolic state termed “Warburg-effect” or “aerobic glycolysis” (Hsu and Sabatini, 2008). Metabolic responses to intracellular bacteria may therefore vary considerably depending on the infected host cell type.

Recently, we reviewed the metabolic adaptations of some important (mainly intracellular) human pathogenic bacteria during intracellular growth (Eisenreich et al., 2010; Fuchs et al., 2012). In the current review, we will focus on the metabolic responses of the host cells to these intracellular bacterial pathogens. For a better understanding of the complex problem, we present in the first part of this review a condensed overview on the major metabolic pathways, nutrient transporters, receptors and regulators controlling the metabolism in mammalian cells (for more details of this aspect, see also Supplementary Material S1–S6), as these metabolic cell processes are potential host cell targets for the interaction with components of the bacterial pathogens.

In the second part, we discuss common and pathogen-specific changes in the host metabolism that are triggered by bacterial pathogens. The focus will be on studies with human and mammalian model hosts or host cells, but insights deriving from alternative infection models (e.g., *Dictyostelium discoideum)* will be included when relevant metabolic data are available.

The interference especially of intracellular bacteria with the phosphoinositide metabolism of host cells which plays a pivotal role in the regulation of receptor-mediated signal transduction, actin remodeling and membrane dynamics of eukaryotic cells will not be included in this review as this topic has been extensively reviewed in the past (Pizarro-Cerdá and Cossart, 2004; Hilbi, 2006; Weber et al., 2009).

## Major metabolic pathways and nutrient transporters of mammalian cells

### Catabolic, anabolic, and anaplerotic pathways

**Figure 2:**
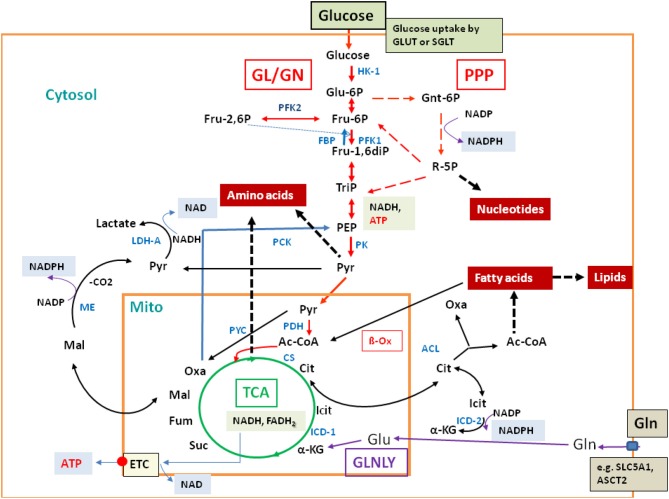
**Major catabolic and anabolic pathways in mammalian cells**. Glucose uptake by the transporters GLUT or SGLT, glycolysis (GL, red arrows) and gluconeogenesis (GN; specific reactions marked by blue arrows); pentose-phosphate pathway (PPP; broken red arrows); tricarboxylic acid cycle (TCA; green circle); glutaminolysis (GLNLY, magenta arrows) and the associated TCA reactions. β-oxidation (β-Ox) and other catabolic reactions occurring in the mitochondrium and (mainly) in the cytosol are marked by black arrows. Anabolic reactions leading to amino acids, nucleotides, and lipids are indicated by broken thick black arrows. Also indicated are the reactions leading to NADH, NADPH, NAD, and ATP, respectively. Metabolites are marked in black and enzymes in blue. Abbreviations: HK, hexokinase; PFK, phosphofructokinase; FBP, fructose bisphosphatase; PK, pyruvate kinase; PDH, pyruvate dehydrogenase complex; PYC, pyruvate carboxylase; PCK, PEP-carboxylase; LDH, lactate dehydrogenase; CS, citrate synthase; ICD, isocitrate dehydrogenase; ACL, ATP-dependent citrate lyase; ME, malate enzyme; ETC, electron transfer chain for aerobic respiration (small red circle), consisting of complexes I–IV and of ATPase (complex V); small blue box: glutamine transporters SLC1A5 and ASCT2.

Glucose and glutamine are the major carbon and/or nitrogen sources for mammalian cells (for reviews, see e.g., Wise et al., 2008; Levine and Puzio-Kuter, 2010). In addition, other carbohydrates and amino acids as well as fatty acids can serve as efficient carbon and/or energy sources. Oxidative degradation of these nutrients occurs via the conserved catabolic pathways [glycolysis (GL), pentose-phosphate pathway (PPP), and the tricarboxylate cycle (TCA)], which are compartmentalized in part to the cytosol and in part to the mitochondria (Figure 2; for more details, see Supplementary Material S1).

Most of the low molecular nutrients, i.e., monomeric carbohydrates, amino acids, fatty acids, and nucleotides, needed for the biosynthesis of proteins, polysaccharides, lipids, and nucleic acids, respectively, are imported from the environment by a large number of membrane-bound transporters (see below). However, if necessary, these cells are also capable of synthesizing the (so-called non-essential) amino acids, fatty acids, purine and pyrimidine nucleotides as well as porphyrines via well-known, highly conserved anabolic pathways. Glucose and other carbohydrates can be synthesized by gluconeogenesis (GN), when nutrition is supported by alternative carbon sources, like glucogenic amino acids, lactate, and glycerol. The essential reactions for GN [from pyruvate via oxaloacetate (OXA) to glucose], are—in addition to the reversible enzymatic GL steps—the reactions catalyzed by pyruvate carboxylase (PC), phosphoenolpyuvate (PEP) carboxykinase (PCK), fructose-1,6-bisphosphatase (FBP), and glucose-6-phosphatase (GP) leading to OXA, PEP, fructose-6-phosphate (F6P), and glucose, respectively (Figure 2).

In contrast to these anabolic pathways, which can occur in most cells, those leading to hormones and bile acids are specific for vertebrates. Synthesis of steroid hormones and bile acids branches-off from the common steroid (cholesterol) biosynthesis pathway, while synthesis of eicosanoid hormones branches-off from the arachidonic acid pathway. The biosynthesis of these compounds requires several cytochrome P450-dependent hydroxylation steps, and the involvement of numerous members of the cytochrome P450 family (encoded by the *CYP* genes). Both classes of hormones participate also—among others—in the regulation of metabolism.

Withdrawal of the TCA intermediates for biosynthetic purposes may lead to the breakdown of the TCA cycle, unless the cycle is replenished in particular by the end products of amino acid catabolic pathways or by specific refilling (“anaplerotic”) reactions (see also Supplementary Material S2).

Glutaminolysis (Figure 2) is an important anaplerotic pathway for refilling the TCA cycle, especially in many cancer cells (Wise et al., 2008). Another major anaplerotic reaction is the generation of OXA catalyzed by the mitochondrial ATP-dependent PC that may be especially relevant during suppression of glutamine metabolism (Cheng et al., 2011).

### Nutrient transporters and nutrient sensors

Most nutrients are channeled into eukayotic cells by solute carrier (SLC) transporters. The human solute-carrier gene (*SLC*) superfamily comprises a large number (at least 362) of putatively functional, protein-coding genes that can be divided into 55 gene subfamilies encoding membrane-bound transporters. They represent passive transporters, symporters, and antiporters, located in all cellular and organelle membranes (possibly except the nuclear membrane). Transported substrates include glucose and other sugars, amino acids and oligopeptides, nucleosides, acetyl coenzyme A, vitamins, fatty acids and lipids, essential metal ions, and other inorganic cations or anions. SLC transporters for the major nutrients may be targets for bacterial pathogens (Lee et al., 2009). Transporter-like sensors (“transceptors”) may be linked to general signaling pathways, such as the protein kinase A (PKA) pathway (Hyde et al., 2003; Holsbeeks et al., 2004). For further details on the elaborate topic of nutrient transporters and nutrient sensors, the reader is referred to Supplementary Material S3, S4 and the specific reviews cited there.

### Metabolic reactions generating antimicrobial agents (ROS and RNI) and reactions balancing the cell-toxic effects of these agents

Mammalian cells [especially professional phagocytes, i.e., monocytes, macrophages, neutrophils, and dendritic cells (DC)] defend themselves against microbial infections not only by specific immune processes, but also by different metabolic reactions as part of the innate immunity. These reactions, mainly catalyzed by the phagocytic NADPH oxidase (PHOX) and the inducible nitric oxide synthase (iNOS or NOS2), respectively, generate reactive oxygen species (ROS) and reactive nitrogen intermediates (RNI), respectively (Bogdan et al., 2000; Nathan and Shiloh, 2000). More details on these enzymes and the regulation of their activity are provided in the Supplementary Material S5.

### The role of autophagy in metabolism

Autophagy is a highly conserved and tightly regulated catabolic process which provides basic metabolites and maintains metabolic homeostasis in cells by degrading long-lived or damaged proteins and organelles (Klionsky, 2007; Rabinowitz and White, 2010). Autophagy is induced by a variety of extra- and intracellular stress stimuli, including nutrient starvation. This dynamic process includes membrane formation and fusion, leading to autophagosome formation, autophagosome-lysosome fusion, and subsequently to breakdown of the autophagosomal contents (which contains many cytoplasmic components) by lysosomal hydrolases. Thus, autophagy is in principle a protective mechanism that sustains cell survival under adverse conditions. Besides maintaining nutrient homeostasis, it is important as defense against intracellular pathogens.

## Regulation of metabolic reactions in mammalian cells

**Figure 3:**
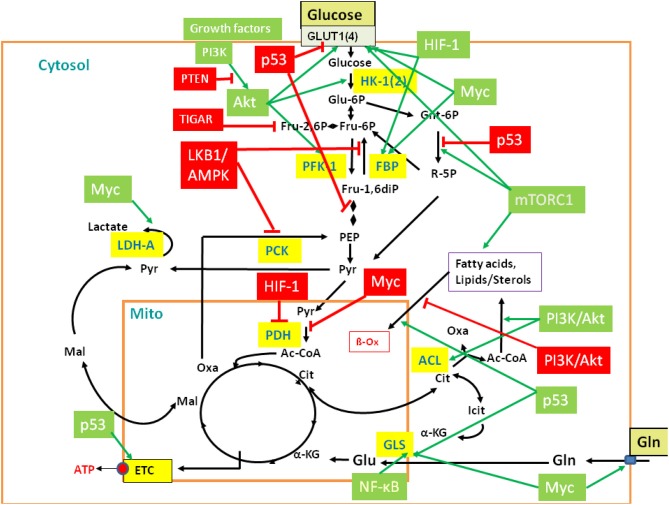
**Regulation of central metabolic pathways by signaling pathways, proto-oncogenes, and tumor suppressors**. The figure provides a rough overview how the major metabolic pathways (GL, PPP, TCA, glutaminolysis, β-oxidation, etc.) are regulated by signaling pathways, protooncogenes, and tumor suppressors, including mainly PI3K/AKT, LKB1/AMPK, HIF-1, mTORC1, MYC, p53, and the p53-controlled regulators TIGAR and PTEN. Interactions between these major players are mediated in part by additional regulatory factors not listed in the figure (Gordan et al., 2007; Levine and Puzio-Kuter, 2010). Red-boxed regulators repress specific metabolic target reactions (indicated by the red bars) whereas green boxed regulators activate specific reactions (indicated by the green arrows). Inhibition and activation of the indicated enzymes may occur on the transcriptional, translational of post-translational level (see text and Supplementary Material S6 for further details). Black arrows indicate the most relevant metabolic steps. Targeted enzymes are yellow-boxed. For abbreviations, see Figure 2 and text.

Uptake and metabolism of carbohydrates (especially glucose), fatty acids, and amino acids (especially glutamine) are controlled by external signals, including hormonal and growth factors [e.g., insulin, glucagon, insulin-like growth factor (IGF), epidermal growth factor (EGF)], external nutrients (e.g., amino acids), and by numerous transcriptional factors (Figure 3). Interestingly, many of the latter factors have been first recognized as tumor suppressors or oncogenes and, hence, their role in cell cycle control, apoptosis, mitochondrial functions, etc., has been extensively studied in the past (but is not discussed here).

In the presence of external glucose, its uptake and flux through the initial reactions of GL are stimulated by the PI3K/AKT pathway. Mitogens and growth factors bind to the growth factor receptors, activate the associated tyrosine kinase which subsequently activates phosphoinositide 3 (PIP3)-kinase (PI3K). PI3K activates protein kinase B (AKT) (Mosca et al., 2011). In particular, PI3K/AKT stimulates expression of the glucose transporter 1 (GLUT1), hexokinase (HK-1), and phosphofructokinase (PFK-1). PI3K/AKT inhibits fatty acid β-oxidation (β-Ox). PI3K also activates the protooncogene c-Myc (MYC) (Dang, 2010) which stimulates glucose uptake, but inhibits flux through the late steps of GL. This provides early glycolytic intermediates for anabolic pathways and supports NADPH production. In addition, activated MYC drives glutamine metabolism. The tumor suppressor p53 (Cheung and Vousden, 2010) inhibits glucose uptake and GL (see below). However, activated AKT phosphorylates Mdm2, the key regulator of p53 stability. Activated Mdm2 ubiquitinates p53 which promotes p53 degradation and thereby stimulates GL. Furthermore, the activated PI3K/AKT pathway enhances translation of the HIF-1α subunit of the hypoxia inducible transcription factor 1 (HIF-1) (Hellwig-Bürgel et al., 2005). This transcription factor also stimulates expression of genes encoding glucose transporters (especially GLUT1), several glycolytic enzymes and lactate dehydrogenase (LDH-A).

On the other hand, signaling through the serine-threonine liver kinase1/AMP-activated protein kinase (LKB1/AMPK) (Shackelford and Shaw, 2009; Hardie, 2011) decreases metabolic flux through GL in response to cell stress. Under low cellular energy conditions (high AMP/ATP ratio), AMPK triggers GL if sufficient external glucose is provided. This results in decreased AMP and glucose levels. Under low glucose condition, sustained activity of AMPK activates p53 which intervenes at several points in GL and oxidative phosphorylation and, in general, slows down GL and promotes oxidative phosphorylation. GL is inhibited by p53-induced repression of the genes for GLUT1, GLUT4, and phosphoglycerate mutase (PGM), while oxidative phosphorylation is enhanced by the increased expression of cytochrome oxidase 2 (SCO2). P53 also induces expression of the “TP53-induced GL and apoptosis regulator” (TIGAR) (Bensaad et al., 2006) and of the “tumor suppressor phosphatase and tensin homolog deleted on chromosome 10” (PTEN) (Liu and Feng, 2012). TIGAR inhibits the glycolytic flux by lowering the fructose-2,6-bisphosphate levels and inhibits expression of the glucose transporters GLUT1 and GLUT4. PTEN blocks PI3K/AKT signaling by dephosphorylation of PIP3. P53 further up-regulates transcription of the *SESN* gene and the produced sestrin proteins stimulate AMPK, eliciting a positive feedback loop. Another p53-activated metabolic target gene is GLS2 which encodes glutaminase 2. This enzyme converts glutamine to glutamate which may enhance the rate of the TCA pathway and oxidative phosphorylation.

AMPK, p53, and AKT converge directly or indirectly on the mammalian target of rapamycin complex 1 (mTORC1) (Dunlop and Tee, 2009). Activated AKT suppresses the tuberous sclerosis complex 2 (TSC2), an inhibitor of mTORC1, thus leading to cell growth while low AKT levels repress growth. On the other hand, activated AMPK stimulates TSC2 leading to inhibition of mTORC1 and hence to reduced cell growth. Figure 3 provides a rough overview of the discussed major players of this complex regulatory network and their metabolic targets.

Translational control elements, e.g., microRNAs (miRNAs), also participate in the regulatory network (Turner, 2000; Klip, 2009; Gao et al., 2009; Vousden and Ryan, 2009; Glatz et al., 2010; Liu et al., 2012; Chen et al., 2012). In addition, metabolic fluxes and regulation of nutrient homeostasis (especially glucose and amino acids) are controlled by post-translational modifications of metabolic enzymes and regulators (mainly by phosphorylation and acetylation) (Xiong and Guan, 2012) and by sharing of metabolic intermediates. Autophagy may also play an important role in regulation of nutrient homeostasis under nutrient starvation conditions by providing essential products for the metabolism. For more details on the regulation of the mammalian cell metabolism, see also Supplementary Material S6.

## Methodological problems related to the analysis of metabolic host cell responses triggered by bacterial pathogens

The significance of infections by bacterial pathogens on the metabolism of the infected host was early recognized (Beisel, 1975, 1984; Richards, 1980). However, conclusions regarding defined changes within the metabolic network of the infected host can hardly be drawn from these studies. The introduction of the high throughput techniques (“omics”) and other modern physical methods made the metabolic aspect of bacterial pathogenesis better accessible. However, as already mentioned above, most of the numerous transcriptome studies on host responses to bacterial infections (for reviews, see Boldrick et al., 2002; Nau et al., 2002; Jenner and Young, 2005; Tam et al., 2008) provide only little information on metabolic changes triggered by the bacterial pathogens in the infected hosts or host cells. In addition, such changes depend to a large extent on post-transcriptional mechanisms, i.e., turnover processes, modulations of enzymatic activities and signaling pathways (e.g., by phosphorylation/dephosphorylation), which are hardly recognized by conventional transcriptome analyses. Application of proteomics for the analysis of metabolic host (cell) responses to bacterial infections—probably better suited for detecting such changes—is still relatively rare. More recently, metabolomics analyzing qualitatively and quantitatively small metabolites in cells or tissue samples of the infected host by MS or NMR have been also applied to determine changes in the metabolite patterns (Han et al., 2008; de Carvalho et al., 2010; Antunes et al., 2011). Other techniques which may provide valuable information on the dynamics of metabolic changes in infected host cells include ^13^C-isotopologue profiling, Raman spectroscopy, nano SIMS, and fluorescence lifetime imaging (FLIM) (Wagner, 2009; Szaszák et al., 2011; Gillmaier et al., 2012; Musat et al., 2012). Each of these methods has, however, specific drawbacks and only the integration of these techniques may provide a satisfactory solution to this complex problem (Zhang et al., 2010).

**Figure 4:**
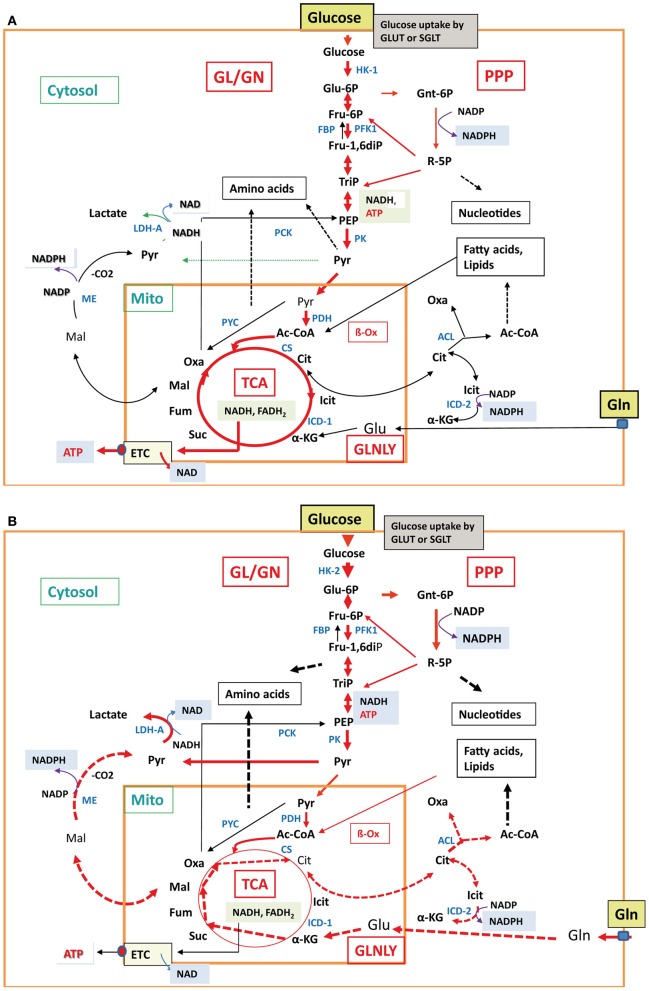
**Major metabolic pathways in normal differentiated cells and cancer cells**. In normal differentiated mammalian cells **(A)**, e.g., epithelial cell or non-activated macrophages, glucose uptake, glycolysis, TCA cycle, and aerobic respiration (indicated by the thick red arrows) are active at low balanced levels. All other less- or non-active catabolic and anabolic pathways are indicated by thin black and broken black arrows, respectively. In mammalian cancer cells **(B)**, carbon metabolism is characterized by enhanced glucose uptake, glycolysis, pentose-phosphate shunt, enhanced conversion of pyruvate to lactate (indicated by the thick red arrows) and reduced conversion to acetyl-CoA by pyruvate dehydrogenase, reduced TCA cycle, gluconeogenesis, and aerobic respiration (indicated by the thin red and black arrows). In some cancer cells, glutaminolysis is also highly induced with the subsequent reactions leading to α-ketoglutarate (α-KG) and malate (Mal) which is converted to pyruvate by the cytosolic malic enzyme (ME) and to citrate (Cit). Cit is transported into the cytosol and converted by ATP-dependent citrate lyase to oxaloacetate (Oxa) and acetyl-CoA (Ac-CoA). These reactions are marked by the thick broken red arrows. Also induced are the anabolic reactions leading to amino acids, nucleotides, and fatty acids/lipids (indicated by the broken thick black arrows). The non-activated metabolic reactions are marked by thin black arrows. For abbreviations, see Figure 2.

Another challenging problem is the choice of “host cells” and “animal models” used in such studies. Many investigations of host cell responses apply established mammalian cell lines, deriving from neoplastic (or artificially transformed) epithelial, fibroblastic, endothelial, or hematopoietic cells. Most differentiated cells perform in the non-activated state a balanced carbon flux through GL, PPP, and the TCA cycle, and oxidative phosphorylation is the main route to generate ATP (Figure 4A). In contrast, most cancer cells use—even in the presence of oxygen—the glycolytic pathway for ATP production, a process known as “aerobic glycolysis” or “Warburg effect” (Warburg, 1956). The initial part of the TCA cycle and oxidative phosphorylation are repressed in these cells. The enhanced GL is accompanied by an increase of glucose uptake and production of metabolic intermediates, needed for increased biomass (nucleic acids, lipids, proteins), characteristic for cancer cells. In addition, enhanced glutaminolysis observed in many cancer cells provides necessary TCA intermediates (Figure 4B). Furthermore, these cells, but also primary macrophages (bone marrow- or blood-derived) used in infection studies, are normally cultured in rich media, containing high levels of glucose, amino acids (especially glutamine) and growth factors. Thus, many cellular nutrient transporters, central pathways involved in carbon and nitrogen metabolism and regulators controlling these processes will be already activated or suppressed (Wise et al., 2008; Levine and Puzio-Kuter, 2010). These metabolic conditions are quite different to those of primary cells encountered by the bacterial pathogens as host cells in *in vivo* infections (Chen and Russo, 2012). Therefore, essential metabolic pathways activated by certain bacterial pathogens in primary cells may not be further activated or even inhibited in cancer cells (or otherwise metabolically activated host cells) by the same pathogens.

The use of protists and invertebrates as infection models may pose another problem for unraveling metabolic host (cell) responses, since metabolic processes of these cells may differ from those of mammalian cells in some aspects, e.g., lack of components of the regulatory network of carbon and nitrogen metabolism. Hence, metabolic responses in these alternative infection models may be quite different from those observed in infected mammalian cells.

## Common and specific metabolic host reactions triggered by bacterial pathogens (general considerations)

Many bacteria (pathogenic and non-pathogenic) trigger metabolic host responses that are primarily directed toward inhibition of survival and proliferation of the bacteria. As shown by numerous transcriptome studies (carried out in established mammalian cell lines, primary phagocytes, or different animal infection models), many of these metabolic responses are unspecific, i.e., they occur with most extracellular and intracellular bacterial pathogens and even with non-pathogenic or killed bacteria and are therefore termed “core host responses” (Boldrick et al., 2002). PHOX generating ROS and the iNOS generating RNI belong to these general metabolic strategies (manifested especially in phagocytic cells), aimed at defending the host against bacteria (Lowenstein and Padalko, 2004; Fang, 2011). Numerous “core response genes” are regulated by NF-κB (Nau et al., 2002). NF-κB is activated (especially in phagocytic cells, an important host cell type in the interaction with bacteria) predominantly by bacterial pathogen-associated molecular patterns (PAMPs, representing mainly common cell envelope components, flagella, CpG DNA motifs, dsRNA) via different Toll-like or NOD-like receptors (TLR or NLR) (Shaw et al., 2008; Kawai and Akira, 2011). Several of the known PAMP-induced NF-κB up-regulated genes encode also enzymes catalyzing important metabolic reactions, including: (a) heme oxygenase-1 (HO-1) which generates carbon monoxide (CO) from heme and thereby protects endothelial cells (EC) from tumor necrosis factor alpha (TNF-α)-mediated apoptosis (Brouard et al., 2002), (b) transporters for Ca^2+^ and other divalent metal ions (ATP2B1; NRAMP2) and for L-amino acids (SLC7A5), (c) adenosine- and adenosine-monophosphate deaminases (ADA, AMPD3), and (d) indolamine-2,3-dioxygenase (IDO).

Furthermore, NF-κB is also involved in control of energy homeostasis and metabolic adaptations by upregulating mitochondrial respiration (Mauro et al., 2011) and glutamine metabolism by decreasing miR-23a expression (Rathore et al., 2012) in a similar manner as Myc. This miRNA represses translation of the mRNA for glutaminase (GLS). In addition, NF-κB seems to participate in the control GLS activity (Erickson and Cerione, 2010).

Major NF-κB down-regulated “metabolic core genes” encode (a) the nucleoside transporter (SLC29A1), (b) enzymes further metabolizing TCA intermediates, like the cytosolic isocitrate dehydrogenase (IDH2) and malic enzyme (ME1), (c) glycogen phosphorylase (PYGL), and (d) the catalytic subunit of glutamate/cysteine ligase (GLCLC) involved in glutathione biosynthesis.

Further common “metabolic” genes are involved in (chole)sterol homeostasis, including the up-regulated gene *HSD11B1* and the down-regulated gene *HSD17B4*. Another apparently general host response to bacterial pathogens is the activation of the transcription factor HIF-1 by stabilization of the HIF-1α subunit through hypoxic conditions caused by infections or pathogen-induced signaling (Rupp et al., 2007; Werth et al., 2010). As described above, HIF-1 controls the transcription of a large number of genes, including genes whose products are involved in glucose uptake and GL.

Together, the metabolic host cell responses triggered by bacterial pathogens that are determined by the “core response genes” aim primarily at increasing antimicrobial activities and decreasing metabolic reactions that may provide essential nutrients to the pathogens, thus inhibiting their proliferation (termed “nutriprive mechanisms”) (Appelberg, 2006).

However, bacterial pathogens may also trigger infection-promoting metabolic host responses that counteract these latter antimicrobial reactions of the host and provide protection and/or increased nutrient supply to the pathogen. These latter host reactions, that are still poorly investigated so far, seem to be induced mainly by pathogen-specific factors. Such metabolic host responses are expected to be different for extracellular and intracellular bacterial pathogens. Extracellular pathogens may influence the host cell metabolism mainly by cell-associated or secreted toxins, adhesins, and exoenzymes or by effector proteins that are translocated into host cells especially via type III or IV secretion systems (e.g., in *Yersinia spp*., enteropathogenic and enterohemorrhagic *E. coli*, *Pseudomonas aeruginosa*). These bacterial compounds may interact with different host cell targets, e.g., components of signaling pathways or transcription factors (e.g., PI3K/AKT; see Figure 3) controlling directly or indirectly host cell metabolism (for reviews see Aktories and Barbieri, 2005; Baldwin and Barbieri, 2005; Aktories, 2011; Dean, 2011).

Intracellular bacterial pathogens may influence (similar to extracellular pathogens) host cell activities prior to invasion already from the outside (Figure 1) by cell-toxic and -adhesive components and/or by effector proteins that are again injected into host cells especially by type III or IV secretion systems (e.g., *Salmonella enterica*, *Legionella pneumophila, Chlamydia spp*; reviewed by Haenssler and Isberg, 2011). Once internalized, these bacteria interfere with the host cell metabolism directly as their proliferation depends on energy sources and metabolites that are either imported or produced by the host cells (reviewed by Eisenreich et al., 2010; Fuchs et al., 2012). The resulting metabolic adaptations of host cells to intracellular bacteria may differ in each case, depending on the specific properties of the two interacting partners. On the part of the bacteria, it is especially the specific metabolic capacities and the specific virulence factors. On the part of the host cells, it is the specific antimicrobial defense mechanisms.

Despite the high relevance of this aspect for the understanding of bacterial pathogenesis, only little information is so far available on changes in the metabolic networks of the major target host cells (e.g., DCs, macrophages, gut epithelial cells, ECs, liver cells) triggered by bacterial pathogens. Since the analysis of metabolic host responses to bacterial pathogens appears to be most advanced for the interaction between mammalian cells and intracellular bacteria, we will focus our discussion in the following on the metabolic host responses triggered by some important human intracellular bacterial pathogens in cell cultures and in *in vivo* infection models.

## Metabolic host responses to selected intracellular bacterial pathogens

### Metabolic host responses to *Salmonella enterica serovar* Typhimurium

*Salmonella enterica* serovar Typhimurium (in the following abbreviated *S*. Typhimurium) is a leading cause of gastrointestinal infections in humans and livestock (Mead et al., 1999; Herikstad et al., 2002). In the mouse model, *S*. Typhimurium leads to systemic infections comparable to *S*. Typhi infections in humans (Tam et al., 2008). The pathogen triggers intestinal inflammation which enhances growth in the intestinal lumen and supports invasion of the intestinal epithelium and mucosal macrophages (Stecher et al., 2007; Santos et al., 2009). After crossing the intestinal epithelium, *S*. Typhimurium is taken up primarily by DCs. Within DCs, salmonellae survive without replication (Niedergang et al., 2000; Jantsch et al., 2003) and are transported to the mesenteric lymph node (MLN). There, they can exit and spread as free bacteria or within cells to other tissues (especially liver and spleen) of the infected mammalian hosts (Paulin et al., 2002; Pullinger et al., 2007).

#### In vitro studies

Numerous “omics”-studies (in particular transcriptome, but also proteome and metabolome analyses) were performed with different host cells and animal (mainly mouse) models infected with *S*. Typhimurium (Rosenberger et al., 2000; Detweiler et al., 2001; Handley and Miller, 2007; Karavolos et al., 2008; Wang et al., 2008; Liu et al., 2010a; Antunes et al., 2011).

Not unexpectedly, transcriptome studies on *Salmonella*-infected mammalian cell lines did not yield much information concerning specific metabolic host responses, i.e., no apparent differential expression of metabolic genes was reported when transcript profiling was performed with *S*. Typhimurium-infected human macrophage-like U937-1 cells (Detweiler et al., 2001). Infected murine macrophage-like RAW 264.7 cells compared to the un-infected control cells showed only up-regulation of *iNOS* and *GLUT1*, encoding NO synthase and Glut1, respectively (Rosenberger et al., 2000). In a proteomic study using RAW 264.7 cells infected with *S*. Typhimurium, increased levels of iNOS and inducible cyclooxygenase 2 (COX2; converting arachidonic acid to prostaglandin) were observed 24 h post-infection (Shi et al., 2009).

#### In vivo studies

*S*. Typhimurium-infected animal models provide more relevant data regarding metabolic host responses. A global differential gene expression study in the mouse colon mucosa during *S*. Typhimurium infection (Liu et al., 2010a) suggests the complete shut-off of the oxidative phosphorylation early in infection (8 h) and the down-regulation of genes of many metabolic pathways, mainly at later times of infection (4 days). Among those, catabolism of glucose and branched chain amino acids (Val, Leu, Ile) seem to be most significantly affected. Interestingly, several genes involved in mTOR signaling are up-regulated.

Cawthraw et al. (2011) analyzed the gene expression profiles induced by *S*. Enteritidis in wild-type C3H/HeN mice and more susceptible TLR4-deficient C3H/HeJ (and BALB/c) mice. In the susceptible mice, more metabolic pathways seem to be affected by the infection than in the wild-type mice, including in particular GL and GN. The response to *S*. Enteritidis in the intestine is, in general, much weaker than the *in vitro* response in murine rectal epithelial cells (CTM93/09 cells), suggesting a tighter control of the metabolic responses to *S*. Enteritidis in the intestine than in the isolated cells (Cawthraw et al., 2011).

An interesting study on metabolic changes in C57BL/6 mice upon *S*. Typhimurium infection was reported (Antunes et al., 2011) using a metabolomics approach. By measuring the altered concentration of metabolites in infected and uninfected intestine and liver, the authors showed that production of many metabolites (including carbohydrates, bile acids, steroids, and eicosanoids among others) are induced or repressed in the samples of the infected mice compared to the uninfected control mice. Most metabolites, derived from primary glucose metabolism (GL, GN, PPP) but also from specific pathways for other carbohydrates (fructose, mannose, galactose, starch, and sucrose), decreased in the liver but not in the intestine upon infection. Whether this finding reflects the high energy expenditure that is required to fight the infection (as suggested by the authors) or a general inhibition of the carbon metabolism in the liver as a result of the *S*. Typhimurium infection remains, however, unanswered. In contrast, a highly increased concentration of metabolites involved in the arachidonic pathway (leading to eicosanoid hormones) and the C_21_-steroid hormone pathway was observed, suggesting that the *S*. Typhimurium infection strongly disturbs the steroid and eicosanoid hormone metabolism of the host. Some of these hormones are known regulators of carbohydrate metabolism and of immune responses (Vinson, 2009).

As mentioned above, activation of HIF-1 due to hypoxic conditions arising in infections by human pathogens seems to be a rather general phenomenon (Werth et al., 2010), which causes increased glucose uptake and GL in most host cells infected by intracellular bacteria. However, hypoxia-independent activation of HIF-1α has also been demonstrated in Peyer's patches of the mice infected by different enterobacteriaceae, including *S*. Typhimurium. The pathogen-specific siderophores (salmochelin in case of *S*. Typhimurium) seem to be responsible for this activation leading to induction of several HIF-1-dependent metabolic genes, including genes for the glycolytic enzymes, hexokinase and phosphofructokinase, as well as carbonic anhydrase, observed in Peyer's patches after orogastric *S*. Typhimurium infection (Hartmann et al., 2008).

*S*. Typhimurium-infected macrophages reduce the host cell's iron pool by causing iron efflux from phagosomes via induced SLC11A1 (NRAMP1) (Cellier et al., 2007; Nairz et al., 2009). Subsequently, the resulting low intracellular iron concentration triggers the expression of the highly efficient iron-uptake systems in *S*. Typhimurium. This allows the bacteria to compete successfully for the remaining iron supply thus promoting their growth within the macrophages. Moreover, strong down-regulation of the gene encoding the plasma membrane calcium-transporting ATPase (*PMCA*) occurs in bovine Peyer' patches early in infection (Santos et al., 2002), thus increasing the cellular calcium level which affects various metabolic processes, including enhanced mitochondrial ATP synthesis (Jouaville et al., 1999).

As described above, PHOX generating ROS and iNOS generating RNI are general metabolic strategies (manifested especially in phagocytic cells) against bacterial pathogens, including *Salmonella*. Silencing of these reactive agents may be therefore advantageous for the pathogen. *S*. Typhimurium is able to subvert these metabolic host defense reactions by different strategies (MacFarlane et al., 1999; Shiloh et al., 1999; Mastroeni et al., 2000). These include: (a) Reduction of the host iNOS-catalyzed RNI production through induction of the arginase isoform II in the spleen of infected mice. The arginase competes with iNOS for the same substrate (arginine) and thus reduces iNOS-mediated NO production which increases the proliferation of *S*. Typhimurium in the lymphoid organs of infected mice (Lahiri et al., 2008); (b) Induction of arginine uptake through enhanced expression of the genes encoding the cationic amino acids transporters (*CAT1* and *CAT2*) in isolated bone marrow-derived macrophages (BMDMs) and DCs, but also in liver and spleen of infected mice (Das et al., 2010). The increased arginine level may lead to an increase of NO production by iNOS and hence to reinforced antimicrobial activity. However, these host cell transporters are recruited preferentially to the *Salmonella*-containing vacuole (SCV) thus channeling arginine into the SCV thereby supporting growth of *S*. Typhimurium. For this goal, *S. Typhimurium* induces in addition the expression of the gene coding for its own arginine transporter ArgT (Das et al., 2010); (c) Using ROS (whose production is induced during *Salmonella* infections due to activated host cell NADPH oxidase) for its own proliferation in the intestinal lumen. ROS trigger the formation of tetrathionate (S_4_O^2−^_6_) from thiosulphate (S_2_O^2−^_3_) enriched in the intestine. Tetrathionate (but not thiosulphate) can be used by *S*. Typhimurium as electron acceptor for efficient anaerobic respiration (Winter et al., 2010).

#### Effector proteins as possible modifiers of host cell metabolism

All *Salmonella enterica* serovars infecting warm-blooded animals express two T3SSs, encoded by SPI-1 and SPI-2, respectively, which translocate specific sets of effector proteins into the host cells cytosol (for a recent review, see Moest and Méresse, 2013). While SPI-1 effectors appear to mainly trigger internalization of *Salmonella* by non-professional phagocytes, the SPI-2 effectors are needed for intracellular replication. Interestingly, recent data suggest that some of these effectors may also be involved in modifying the metabolism of the infected cells.

As shown (Knodler et al., 2005), *S*. Typhimurium residing in the SCV translocates SopB which activates Akt. This activation occurs within minutes after invasion and lasts for several hours. The Akt activation seems to be involved in the inhibition of apoptosis of the infected host cells (Knodler et al., 2005) and is essential for *Salmonella* (and *Mycobacterium tuberculosis*) infection of epithelial cells and macrophages (Kuijl et al., 2007). As discussed above, activated Akt phosphorylates Mdm2, a key regulator of p53 stability. Activated Mdm2 ubiquitinates p53 and thus promotes p53 degradation which may in turn stimulate glucose flux via GL.

AvrA, another *Salmonella* T3SS-translocated effector protein (Hardt and Galán, 1997), could also be responsible—either directly or indirectly—for some of the specific *Salmonella*-induced metabolic host responses. As shown (Liu et al., 2010b), AvrA leads to reduction of oxidative phosphorylation and to activation of mTOR, NF-κB and p53 in intestinal *in vivo* infection. The activation of p53 is caused by AvrA-mediated p53 acetylation (Wu et al., 2010). While activated p53 will inhibit GL, activated mTOR may stimulate amino acids uptake and catabolism. Thus, the AvrA-mediated activation of these regulators may again modify the carbon metabolism of *Salmonella*-infected host cells.

In a recent study, Lopez and collegues showed that *S*. Typhimurium strains expressing the type III effector protein SopE (encoded by a lysogenic phage present in several epidemic strains) triggers the synthesis of iNOS in the host cells intestine (Lopez et al., 2012). The thereby produced nitric oxide is converted to nitrate in the host cell which acts as an even better electron acceptor in anaerobic nitrate respiration than S_4_O^2−^_6_ in tetrathionate respiration. Under these respiratory conditions, *S*. Typhimurium is able to use the non-fermentable carbon source ethanolamine (Thiennimitr et al., 2011), a nutrient deriving from phosphatidylethanolamine that is present in the intestine (Bertin et al., 2011). This metabolic capability generates a growth advantage for *S*. Typhimurium over the inherent anaerobic intestinal microbiota that is unable to respire ethanolamine.

*Salmonella* infection of murine macrophage-like cell lines (RAW264.7 and J774A.1) induces the expression of *COX2* (Uchiya and Nikai, 2004; Shi et al., 2009). This induction depends on the *Salmonella* protein SpiC, a gene product encoded within SPI-2 and translocated into host cells by T3SS-2 (Uchiya and Nikai, 2004). *COX2* encodes cyclooxygenase 2, a key enzyme involved of prostanoid synthesis which leads to an increased production of PGE_2_ and PGI_2_, two prostanoids belonging to the eicosanoid hormones.

In summary, a plethora of *in vitro* and *in vivo* studies shows—albeit with still rather fragmentary evidence—that *Salmonella* infection leads to numerous changes of the carbon and nitrogen metabolism in the infected host (cells), which on one hand may prolong the host cell's survival and on the other hand may support the pathogen's proliferation. These metabolic changes of the host (cells) are caused by common and specific components of the pathogen. Especially the latter host cell responses are observed in *in vivo* models, but hardly in established cell lines. Multiple-task effector proteins, e.g., SopB, AvrA, SopE, may also be responsible for the specific changes in host cell carbon and nitrogen metabolism, by interfering with global regulators of the host cell's metabolism, like p53 and mTOR. Clearly, further studies are necessary to fully understand the impact of *Salmonella* infection on the complex metabolic network of the host and to unravel which *Salmonella* effectors are responsible for the observed specific metabolic changes of the host cells.

### Metabolic host responses to *Mycobacterium tuberculosis*

The facultative intracellular pathogen *M. tuberculosis* belongs to the few bacterial pathogens for which humans are the only known reservoir, although it may infect mice and even amoebae (Hagedorn et al., 2009). *M. tuberculosis* colonizes the lung as major target organ, causing pulmonary tuberculosis (TB). Macrophages are the primary intracellular niches to which *M. tuberculosis* has adapted. Within these host cells, *M. tuberculosis* survives and replicates in a specialized phagosomal compartment. Under certain conditions, the bacteria seem to be able to escape into the host cells cytosol (van der Wel et al., 2007). Shortly after infection, granuloma are formed comprising a core of infected macrophages, surrounded by foamy macrophages, monocytes, and multinucleated giant cells (Russell, 2007). Under immunocompromised conditions, *M. tuberculosis* may also infect other organs, causing extrapulmonary TB. The visceral adipose tissue has been proposed as an important reservoir for persistence of *M. tuberculosis* (Neyrolles et al., 2006).

Because of its medical importance, extensive effort has been made to understand the complex metabolism of *M. tuberculosis* under *in vitro* and *in vivo* conditions. This aspect is far from being completely solved (Rhee et al., 2011), but the metabolic responses of the target host cells and tissues to *M. tuberculosis* infections are even less understood. Numerous studies using transcript profiling were performed with infected host cells (mainly macrophages) and mice (Ehrt et al., 2001; Wang et al., 2003; Kendall et al., 2004; Volpe et al., 2006). In addition, proteomics and metabolomics studies were applied to *M. tuberculosis*-infected cells and animal models (Shui et al., 2009; de Carvalho et al., 2010; Somashekar et al., 2011, 2012). These studies allowed important insights into immune responses induced by *M. tuberculosis*, but also provided some clues how *M. tuberculosis* modulates metabolic processes of the infected host cells.

#### In vitro studies

Most of the observed metabolic changes in *M. tuberculosis*-infected host cells are again linked to defense mechanisms, including oxidative stress and production of antimicrobial peptides. Macrophages can inhibit *M. tuberculosis* replication by iNOS-generated RNI (Adams et al., 1997). This enzyme is induced upon *M. tuberculosis* infection of macrophages (Ehrt et al., 2001). Induction of PHOX, leading to increased levels of ROS has also been reported (Wang et al., 2003; Shui et al., 2009). Yet, *M. tuberculosis* is relatively resistant to killing by ROS as these ROS are inactivated by the induced mycobacterial catalase KatG (Ng et al., 2004) which has catalase and peroxidase activity (Heym et al., 1993). *M. tuberculosis* infection also stimulates expression of heme oxygenase (HO-1) in mouse macrophages and other host cells, probably via the TNF-α signaling pathway (Shiloh et al., 2008). CO, one of the reaction products of HO-1, together with NO generated by iNOS, seems to induce transcription of the Mtb dormancy regulon via the two-component system DosS/T (Shiloh et al., 2008). Furthermore, increased levels of host cell proteins involved in the generation of ROS, like the p67phox subunit of NADPH oxidase and the neutrophil cytosolic factor 1 (NCF1 or p47) required for activation of NADPH oxidase are observed. Finally, the production of the Mn-dependent superoxide dismutase, acting as an antioxidant (by quenching ROS and hydrogen peroxide) is up-regulated.

The unique mycobacterial cell wall lipids play an important role in *M. tuberculosis* pathogenesis processes (Russell et al., 2002; Korf et al., 2005). Exposure of J774A.1 macrophage-like cells to *M. tuberculosis* lipids results in a total of 166 differentially expressed macrophage proteins (Shui et al., 2009). A substantial portion of the differentially expressed proteins (14%) seem to be involved in metabolism, but the functions of these proteins are not further elaborated.

#### In vivo studies

Progression of human TB leads to the development of caseous pulmonary granuloma, comprising a core of infected macrophages. Previous reports show an abundance of lipid species (mainly cholesterol, cholesterol ester, and triacylglycerol) in these cells of *M. tuberculosis*-infected mice and TB patients (Garner et al., 2002; Hunter et al., 2007). Transcriptome analysis of such TB granuloma reveals significant up-regulation of genes involved in sequestration, catabolism, and synthesis of host lipids (Kim et al., 2010). Many of the up-regulated genes are also induced by TNF-α, indicating that this response may be caused by the sustained inflammation triggered mainly by *M. tuberculosis* cell wall components. The thereby accumulated lipids could serve as suitable carbon source for *M. tuberculosis* colonizing the granuloma in a dormant state.

Important information on metabolic host changes is provided by recent ^1^H-NMR-based metabolite profiling studies from the mouse and guinea pig *M. tuberculosis* infection models (Shin et al., 2011; Somashekar et al., 2011). The infected mice show qualitatively similar changes of major catabolic and anabolic metabolites in all tissues tested (lung, liver, and spleen). Quantitatively, the most significant effects are observed in the lung, the major target of *M. tuberculosis* infection. The levels of glucose and glycogen as well as those of NAD and NADP decrease, while lactate concentration increases, suggesting increased consumption of glucose via GL and the pentose-phosphate shunt. The decreased level of the TCA cycle intermediates OXA and fumarate, suggesting reduced activity of the TCA cycle, supports this assumption. Yet, the level of succinate, another TCA cycle intermediate increases. This elevated amount of succinate may be generated by enhanced glutaminolysis in mitochondria induced by the oxidative stress occurring during *M. tuberculosis* infection (see above) (Fedotcheva et al., 2006). Alternatively, succinate may be secreted by *M. tuberculosis*. As shown (Muñoz-Elías and McKinney, 2005), bacterial production of succinate could increase due to the induction of the glyoxylate and methylcitrate cycles caused by switching the *M. tuberculosis* carbon metabolism toward β-oxidation of fatty acids due to the enhanced lipolysis in the host cells. Interestingly, the amount of many amino acids also increases in the analyzed tissues and even in serum of the *M. tuberculosis*-infected mice, suggesting enhanced proteolysis and/or catabolism of ingested amino acids. This metabolic change might be related to the “anabolic block” observed in TB patients (Macallan et al., 1998).

Furthermore, increased amounts of several intermediates of pyrimidine and purine nucleotide biosynthesis are observed in the lung which is taken as evidence that cells in the *M. tuberculosis*-infected lung actively divide (Shin et al., 2011). An increased level of the antioxidant glutathione (GSH) is also observed in the infected lung (and spleen) and probably represents an additional metabolic defense reaction against the induced oxidative stress. Metabolite profiling of the lung tissues and serum of infected guinea pigs (Somashekar et al., 2011) reveal similar patterns of metabolites as in the infected mice, also suggesting enhanced GL, glutaminolysis and GN triggered by the *M. tuberculosis* infection.

In summary, *M. tuberculosis* infection causes strong metabolic host responses concerning host defense against the infection, but also enhanced lipid metabolism, glucose consumption, and proteolysis. These metabolic host responses, observed again mainly in the *in vivo* (mouse and guinea pig) models, seem to support intracellular replication of *M. tuberculosis*. The unique mycobacterial cell wall components apparently play a major role in most of the observed metabolic host responses.

### Metabolic host response to *Listeria monocytogenes*

The Gram-positive facultative intracellular bacterium *L. monocytogenes* is the only species of the genus *Listeria* that is pathogenic for humans, whereas the other members are either pathogenic only to animals (*L. ivanovii*) or are harmless saprophytes living mainly in natural environments. Clinical symptoms of *L. monocytogenes* infections range from febrile gastroenteritis to encephalomeningitis, sepsis, and abortion. *L. monocytogenes* escapes from the phagosome and replicates primarily in the host cells cytosol, but replication in so-called spacious *Listeria*-containing phagosomes (SLAPs) may also occur (Birmingham et al., 2008). The latter mode of intracellular listerial replication is dependent on the recruitment of the autophagy protein LC3 to the phagosome and low production of listeriolysin (LLO). The mechanism of action of this and the other virulence factors of *L. monocytogenes* has been extensively studied on the molecular level using appropriate cellular and animal model systems (for recent reviews, see Camejo et al., 2011; Stavru et al., 2011). The expression of most of these virulence factors is induced within the infected host cells (reviewed by de las Heras et al., 2011).

#### In vitro studies

Genes involved in metabolism (with the exception of *HIF-1*α) are apparently not differentially expressed in the human macrophage-like cell line THP-1 upon *L. monocytogenes* infection (Cohen et al., 2000). This result may be in part the consequence of the incomplete gene array used in this study, but—more likely—is again due to the already upregulated metabolism of the used THP-1 cells (even in the uninfected state—see above). This assumption is supported by recent ^13^C-isotopologue studies, carried out with primary murine bone marrow-derived macrophages (BMM) and the murine macrophage-like J774A.1 cells infected with *L. monocytogenes* (Gillmaier et al., 2012). When the two cell types are fed with uniformly ^13^C-labeled glucose or ^13^C-labeled glutamine, high induction of glucose uptake and GL is observed in BMM upon infection with *L. monocytogenes*, but not in the J774A.1 cells (which already show up-regulated GL and glutaminolysis in the un-infected state). In contrast, the activity of the TCA cycle and glutaminolysis remains unaffected in the *L. monocytogenes*-infected BMM. The *L. monocytogenes*-induced carbon metabolism in BMM allows a similarly efficient proliferation of the internalized bacteria in BMM as in J774A.1 cells.

#### In vivo studies

In mice expressing humanized E-cadherin, the host response was analysed by transcript profiling of the intestinal epithelia after infection with a *L. monocytogenes* wild-type strain and compared to the response of germ-free mice and of mice infected with a non-pathogenic *L. innocua* strain (Lecuit et al., 2007). The supplementary part of this report provides interesting data concerning specific *L. monocytogenes*-induced metabolic host responses. Transcription of most GL genes is enhanced after infection, but the gene encoding hexosekinase II (*HKII*) shows the highest increase (about 10-fold). This suggests that glucose taken up is mostly phosphorylated by HK-II, which has easy access to ATP due to its close association to mitochondria. The product, glucose-6P, is an important carbon substrate for intracellular *L. monocytogenes* in epithelial cells and macrophages (Chico-Calero et al., 2002; Eylert et al., 2008). The enhanced glucose-6P production will be therefore advantageous for the cytosolic proliferation of *L. monocytogenes*. The induced expression of the GL genes and of the gene for lactate dehydrogenase may be linked to the observed enhanced *HIF-1*α gene expression. In line with the increased expression of the GL genes is the down-regulation of the genes for fructose bisphosphatases 1 and 2 (playing a central role in GN) and of glutamate dehydrogenase (involved in glutaminolysis). Transcription of the genes encoding pyruvate dehydrogenase and citrate synthase is also increased while all other genes encoding TCA cycle enzymes and the genes involved in the electron transfer chain (ETC) are not differentially regulated. Possibly, the produced citrate is mainly transported into the cytosol and converted by ATP-dependent citrate lyase into OXA and acetyl-CoA and used for biosynthesis of aspartate and fatty acids, respectively. There is also a remarkable up-regulation of genes encoding key enzymes involved in the biosynthesis of serine (phosphoserine aminotransferase) and asparagine (asparagine synthetase). The observed metabolic host responses appear to be triggered by intracellular listeriae since a non-invasive *inlA, inlB* mutant does not cause up-regulation of these genes. This suggests that secreted listerial virulence factors whose synthesis is induced under intracellular conditions may modulate the metabolic host responses observed in this *in vivo* study and also in the above described ^13^C-isotopolog studies with *L. monocytogenes*-infected primary mouse macrophages. The two phospholipases C (PlcA and PlcB) and listeriolysin are potential candidates, as they were shown to interact with signaling pathways of mammalian cells (Goldfine and Wadsworth, 2002; Kayal and Charbit, 2006; Gekara et al., 2008).

Another notable result of this *in vivo* transcriptome analysis is the dramatic down-regulation of several *CYP* genes encoding members of the cytochrome P450 families which represent monooxygenases catalyzing mainly the hydroxylation of steroid and eicosanoid substrates (Marohnic et al., 2006). The *CYP* genes that are most strongly down-regulated in the *L. monocytogenes*-infected mouse intestinal epithelium encode monooxygenases involved in biosynthesis of oxysterols and bile acids (Pikuleva, 2006). In this context, recent data (Zou et al., 2011) is of interest showing that *L. monocytogenes* triggers the up-regulation of *Ch25h* in macrophages. This gene encodes cholesterol 25-hydroxylase responsible for the formation of 25-hydroxycholesterol from cholesterol. Increased levels of Ch25h support survival of *L. monocytogenes*-infected cells (Zou et al., 2011). In accord with this assumption is the strong down-regulation of *CYP39A1* encoding a hydroxylase that converts cholesterol via the 24-cholesterol pathway and thus reduces the formation of 25-hydroxycholesterol.

As expected, transcriptional up-regulation of the genes encoding iNOS and IDO (indolylamine 2,3-dioxygenase) is also observed which in conjunction with the transcriptional down-regulation of the genes for arginase type II (competing with iNOS for Arg) and catalase supports host defense reactions against *L. monocytogenes*. This finding is in accord with results of another study (Sonje et al., 2010) also showing highly increased expression of *iNOS* and *IDO* in neonatal mice upon infection with *L. monocytogenes*. It should be noted, however, that induced expression of these genes occurs even in TNF receptor 1 (TNFR1) knock-out mice, which are highly susceptible for *L. monocytgenes* infection. This indicates that increased levels of iNOS and IDO do not suffice to cause antilisterial protection and that TNF-α signaling is absolutely needed for protection against *L. monocytogenes*.

In summary, *L. monocytogenes* has a strong impact on the host cell metabolism, especially when internalized by mammalian host cells. Some of the virulence factors, notably LLO and the two phospholipases, may be responsible for at least some of the observed metabolic host responses. Especially the studies with *L. monocytogenes*-infected “humanized” mice and with primary mouse macrophages show that *L. monocytogenes* induces reactions of the host cell carbon metabolism that favor uptake of glucose and the production of compounds, such as glucose-6P, serine, and glycerol, which are preferably utilized by the bacteria for their own intracellular metabolism (Eylert et al., 2008). Furthermore, the internalized bacteria appear to trigger a striking change of the host cell steroid metabolism that may also support intracellular survival (e.g., by causing LLO activation, and/or reduced bile acid formation).

### Metabolic host response to *Chlamydia spp*.

The genus *Chlamydia* comprises several obligate intracellular species. Among those, *C. trachomatis* is the most extensively studied. In humans, *C. trachomatis* infects ocular and urogenital epithelial tissues and causes trachoma and sexually transmitted diseases (Byrne, 2010). All *Chlamydia* species exhibit a partially disrupted primary carbon metabolism and lack most anabolic pathways. Hence, intracellular chlamydiae have to take up most of the required metabolites and energy from the host cell (McClarty, 1994; Fuchs et al., 2012). Amino acids seem to be the major carbon and energy source for the impaired metabolic activities of the intracellular chlamydiae. Amino acids may be provided to the bacteria by the host cell through lysosomal degradation of external proteins (Ouellette et al., 2011). In addition, the chlamydial proteases CPAF and cHtrA are secreted into the host cells cytosol where they may degrade host proteins (Zhong et al., 2001; Wu et al., 2011; Chen et al., 2012). For sustaining a stable host-parasite interaction, the host cell has to substantially re-adjust its metabolism upon infection with chlamydiae.

#### Studies with Chlamydia-infected host cells and tissues

Since there are no suitable animal models for *Chlamydia*, all gene expression profiling studies reporting on host cell responses to chlamydial infections are performed with infected cell lines and tissues (e.g., Stephens, 2003; Alvesalo et al., 2008; Amirshahi et al., 2011). Not unexpectedly, these studies provide only little information on differentially expressed metabolic genes. In Hep-2 cells infected with *C. pneumoniae*, induction of host cell genes involved in glucose uptake and GL is observed, caused (at least in part) by hypoxia-mediated HIF-1α stabilization (Rupp et al., 2007; Werth et al., 2010).

Host-cell derived lipids, especially sphingomyelin, are essential for intracellular growth of *C. trachomatis* and the formation of the inclusion body. Reactivation of persistent *C. trachomatis* requires the induced host cell sphingolipid biosynthesis (Robertson et al., 2009). The enhanced lipid biosynthesis in the host cells relies on higher NADPH consumption which may explain the preferential glucose flux through the PPP observed in *C. trachomatis*-infected cells (Fukuda et al., 2005; Szaszák et al., 2011).

A recent gene expression analysis in conjunctival swab samples of patients with active trachoma and healthy controls (Natividad et al., 2010) provides (mainly in the Supplementary Material part) a plethora of interesting data on differentially expressed metabolic genes that may shed light on the metabolic host responses to *C. trachomatis* infection. Of particular note is the highly up-regulated transcription of many genes encoding nutrient transporters, i.e., transporters for glucose, including members of the SLGT family (e.g., SLC5) and the GLUT family (e.g., SLC2A3, SLC2, SLC2A14, SLC2A5), for cationic, neutral, and aromatic amino acids (e.g., SLC7A11, SLC14A4, SLC16A, SLC1A4, SLC7A11), for peptides (SLC15A3), for metal ions (e.g., SLC39A8, SLC8A1, SLC24A, SLC11A1), for glycerol-3P (SLC37A) and for nucleosides (SLC38A3). Assuming a correspondingly increased translation of these transcripts, the data suggest that induction of transporters required for the uptake of nutrients essential for intracellular growth is a strong metabolic host response to a chlamydial infection. It will be interesting to find out whether these transporter proteins are incorporated into the host cell cytoplasmic membrane or recruited to the *Chlamydia* containing vacuole (“inclusion body”). Not surprisingly, none of these transporter genes upregulated in the tissue samples have been found differentially expressed in productively and persistently *Chlamydia*-infected HeLa or HL cells using transcriptomic (Eickhoff et al., 2007; Alvesalo et al., 2008) or proteomic approaches (Savijoki et al., 2008). As discussed above, nutrient uptake is already induced in these transformed cell lines. Indeed, expression of amino acid transporter genes and of other metabolic genes seems to be even down-regulated in these transformed host cells upon infection with *C. pneumoniae* (Alvesalo et al., 2008).

As expected, increased expression of genes encoding NADPH oxidase and iNOS is also observed in the above gene expression analysis of the patients' tissue samples. This causes enhanced production of ROS and RNI which may support the host defense against the intracellular *Chlamydia*. The enhanced expression of the host genes for superoxide dismutase (*SOD2*) and arginase II (*ARG2*) probably protects the host cells against the cell-damaging ROS and RNI.

In summary, the metabolic host cell responses to chlamydial infections are highlighted by increased glucose uptake and glucose flux through GL. The pentose-phosphate shunt is also induced and may be linked to the enhanced anabolic performance (increased protein and lipid synthesis) of the host cells observed during the proliferative phase of the chlamydial infection. The remarkable up-regulation of many genes encoding transporters for nutrients needed for intracellular chlamydial growth suggests increased import of these nutrients by the host cells and possible recruitment of these nutrient transporters to the membrane surrounding the *Chlamydia*-containing vacuole (“inclusion”). So far, nothing is known about chlamydial factors that may be responsible for the metabolic responses of the infected host cells.

### Metabolic host response to *Coxiella burnetii*

*C. burnetii*, the etiological agent of the human disease Q fever, belongs—like the *Chlamydia spp*.—to the group of obligate intracellular human pathogens, but replicates in acidified (pH 4.5–5) phagolysosome-like vacuoles.

Transcriptional host response to *C. burnetii* infection is mainly studied in the human macrophage-like cell line THP-1 (Ren et al., 2003; Mahapatra et al., 2010). THP-1 cells, infected with *C. burnetii* or *Chlamydia trachomatis*, respectively, show an extensive overlap in up- and down-regulated genes, but pathogen-specific differential gene expression is also observed (Ren et al., 2003). Only few of the common and species-specific differentially regulated genes are involved in host cell metabolism and none in carbon metabolism which may be again due to the already activated primary carbon metabolism of the host cell line used (see above).

The common up-regulated genes include *SLC2A6*, encoding a facilitated transporter that can also transport glucose, *TDO2*, encoding tryptophan 2,3-dioxygenase which, like indoleamine 2,3 dioxygenase (IDO), catalyzes the first step in the degradation of tryptophan, *CYBB* (*NOX2*), encoding gp91, the major subunit of phagosomal NADPH oxidase, and *FTH1* (encoding ferritin heavy chain) which oxidizes Fe(II) to Fe (III). In addition to the transcriptional up-regulation of *FTH1*, *C. burnetii*, unlike *C. trachomatis*, also induces the expression of the transferrin receptor gene (*TfR*) in murine macrophage-like J774A.1 cells, resulting in a 2.5-fold increase in cellular iron concentration (Howe and Mallavia, 1999). Since the metabolic activities of *C. burnetii* are strongly affected by iron *in vitro*, this finding suggests that the *TfR* up-regulation is important for the iron supply during intracellular replication of *C. burnetii*. In addition, the reactions catalyzed by NOX2 and NADP oxidase may help to protect the host cells against exceeding growth of the intracellular bacteria.

Among the host metabolic genes that are specifically up-regulated by *C. burnetii* are *RENBP* encoding N-acylglucosamine 2-epimerase that is involved in amino sugar and nucleotide sugar metabolism, *PIR* (Wendler et al., 1997) encoding the iron-binding protein and transcriptional co-factor pirin, which is involved in numerous cellular processes, and *ATP6V1H* encoding a subunit of the V-ATPase, a vacuolar proton pump.

Changes in cellular lipids, especially in the cellular cholesterol level, have been observed during infections by phagosomal intracellular bacteria, including *S. enterica*, *M. tuberculosis*, and *C. burnetii* (Howe and Heinzen, 2006). The up-regulation of host genes involved in lipid metabolism in *C. burnetii* infected THP-1 cells (Ren et al., 2003; Mahapatra et al., 2010) is in line with this observation.

Although both, ROS and RNI are involved in the host defense against *C. burnetii* infection (Brennan et al., 2004), a hallmark in metabolic host response to *C. burnetii* is the dramatic decrease of NADPH oxidase in neutrophils (Siemsen et al., 2009) which seems to be due to the inactivation of neutrophil NADPH oxidase by phagocytosed *Coxiella*. A T2SS-dependent secreted acidic phosphatase of *Coxiella* appears to be responsible for this process (Hill and Samuel, 2011). Bacterial phosphatases could be even more generally involved in silencing the NADPH oxidase by intracellular bacteria, as these bacteria frequently encode secreted protein- and/or phosphoinositide-phosphatases (Tabernero et al., 2008; Kastner et al., 2011).

*C. burnetii* also secretes protein effectors into the cytosol via a T4SS that is similar to the Dot/Icm T4SS of *Legionella pneumophila* (Carey et al., 2011). As in *L. pneumophila* (see below), effector proteins have been described that modulate host cell functions, e.g., membrane trafficking (Voth and Heinzen, 2009), but so far no *Coxiella* effector has been identified that might be involved in the modulation of host cell metabolism.

In summary, the scattered information on the metabolic host response against *Coxiella* infection is mainly based on transcriptome data obtained from *Coxiella*-infected cell lines and is hence of limited significance, especially as far as the primary carbon metabolism is concerned. Major host metabolic responses seem to be the induction of iron uptake and the destruction of the NADPH oxidase caused by *Coxiella* infection, two reactions that favor the intracellular replication of these bacteria.

### Metabolic host response to *Legionella pneumophila*

*L. pneumophila* is the best studied member of the genus *Legionella*. This facultative intracellular pathogen replicates intracellularly within a specialized phagosome, the *Legionella*-containing vacuole (LCV) in human alveolar macrophages and in amoebae. *Acanthamoeba castelanii* and the social amoeba *Dictyostelium discoideum* are well-established model organisms for studying the *L. pneumophila* intracellular life style. Virulence of *L. pneumophila* is most strikingly determined by the Dot/Icm T4SS, which translocates about 300 effector proteins into the host cell (Isberg et al., 2009). Some of these effectors participate in the remodeling of the primary phagosome to the LCV, essential for intracellular replication, and in the manipulation of host cell signal transduction pathways through phosphorylation/dephosphorylation reactions (for recent reviews, see Shin and Roy, 2008; Haenssler and Isberg, 2011).

An explicit participation of translocated effectors in the modulation of the host cell's metabolism has not yet been demonstrated. However, some observations argue for a direct or indirect involvement of effector proteins in metabolic processes: (a) in macrophages and in the *Dictyostelium* model, Dot/Icm effectors may play a critical role in autophagy with a possible consequence for host cell metabolism (Otto et al., 2004; Amer et al., 2005; Tung et al., 2010), (b) *L. pneumophila*-infected human macrophage-like U937 cells activate NF-κB signaling in a Dot/Icm-dependent manner (Losick and Isberg, 2006) by the effector protein LnaB (Losick et al., 2010). NF-κB, well-known as an important regulator of cell survival and differentiation, is also linked to metabolic processes, e.g., glutamine metabolism (Rathore et al., 2012), (c) putative Dot/Icm-dependent serine/threonine protein kinases and phosphatases may modulate the activity of metabolic enzymes as well as host signaling pathways and possibly downstream metabolic processes (Chien et al., 2004; Li et al., 2009; Hervet et al., 2011).

Comprehensive transcriptome studies on host cell response to *L. pneumophila* infection are so far only available for *L. pneumophila*-infected *D. discoideum* (Farbrother et al., 2006). Differential expression is observed for many metabolic genes of *D. discoideum* (mainly seen 24 h after infection) whose impact on host cell metabolism is summarized by the authors in two major conclusions: (a) enzymatic activities involved in bacterial degradation, protein biosynthesis, and fatty acid modification are down-regulated and (b) expression of genes involved in metabolic activities that ultimately generate products needed for the proliferation of the pathogen is induced. Based on the data, the authors suggest that “*Legionella* specifically manipulates the host by differentially regulating its metabolism, upregulating activities that produce nutrients suitable for the pathogen and downregulating the host-specific metabolism.”

This rather general statement is in line with a more in depth biochemical study (Wieland et al., 2005) showing that expression of the amino acids transporter SLC1A5 (specific for neutral amino acids) is highly induced in the human monocyte cell line MM6 upon infection by *L. pneumophila*. This study also shows that functional SLC1A5 transporter is important for *L. pneumophila* proliferation in these cells and preliminary evidence suggests that SLC1A5 is translocated to the LCV membrane thus supporting the uptake of cysteine, serine, and glutamine into the LCV. These amino acids seem to be essential carbon sources for growth of intracellular *L. pneumophila* (Price et al., 2011). Interestingly, induction of the amino acid transporter SLC1A5 has been also reported in THP-1 cells infected by the intracellularly replicating *Francisella tularensis* (Barel et al., 2012).

In summary, solid experimental data on metabolic responses of the primary host cells (i.e., macrophages in human infections and amoebae in natural environments) to *L. pneumophila* are scarce. Clearly, in depth biochemical studies (especially using human macrophages) are needed to support the interesting, albeit mostly still preliminary findings, and to answer the important question of how *L. pneumophila* modulates the metabolism in its target cells. Some of the numerous *Legionella* effector proteins might be interesting candidates.

## Conclusions

Despite the rather limited knowledge that is currently available concerning metabolic responses of mammalian cells to bacterial pathogens, some common and pathogen-specific motifs are recognizable. As expected, most of the general observed changes in host cell metabolism support cell protection and include in particular the induced expression of *iNOS*, *PHOX*, and *IDO*, leading to increased production of ROS, RNI, and tryptophan degradation products, respectively. The also frequently observed decreased expression of nucleoside transporter, isocitrate dehydrogenase, malic enzyme, and PYGL may cause reduced access to essential metabolic intermediates for the invading pathogens. Another common theme seems to be the generation of hypoxic conditions leading to HIF-1α stabilization with the subsequent induction of HIF-1 dependent genes which comprise also the genes involved in glucose uptake and GL. These common metabolic host responses (denoted as “core response”) are mainly triggered by bacterial PAMPs, shared by pathogenic and non-pathogenic bacteria.

However, bacterial pathogens may induce in addition specific metabolic host responses which are probably in favor of these bacteria. As illustrated by the intracellular pathogens discussed above (most information on metabolic host responses comes from intracellular bacterial pathogens), some evidence exists that specific virulence factors and effector proteins may trigger responses that divert metabolic host reactions in support of the survival and growth of the individual pathogen. However, there is still little knowledge on the precise nature of these bacterial factors and even less on the interacting host targets causing the specific metabolic host changes.

**Figure 5:**
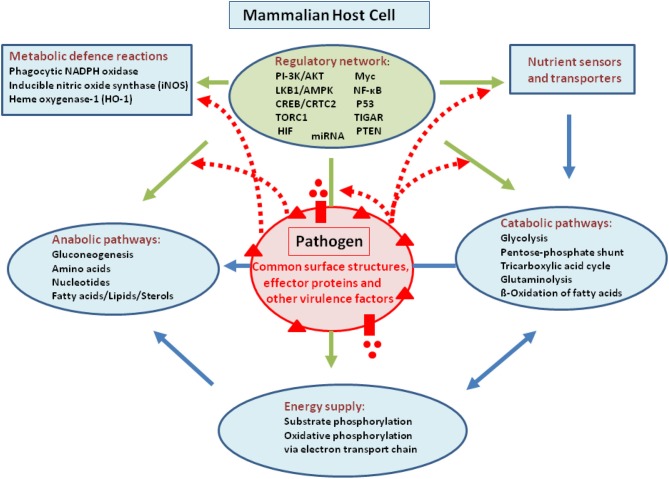
**Summary overview showing the major metabolic pathways and reactions of a metabolically active mammalian host cell (blue spheres and boxes) and the regulatory network with the major regulators (green box) that control host cell metabolism**. Blue arrows indicate exchange of metabolites and intermediates of the different metabolic functional units. Green arrows indicate control of these functional units by specific regulators. The pink sphere indicates an (intracellular) bacterial pathogen with surface components (red triangles), T3SS or T4SS effector proteins and other secreted virulence factors (red bars and circles) that might interact with metabolic targets of the host cell. The red dashed arrows indicate already confirmed or probable interactions (see text for details).

The so far recognized specific host responses triggered by bacterial pathogens include: (a) induction of host cell enzymes which dampens the production or action of ROS and RNI, e.g., catalase and arginase, (b) further enhanced glucose uptake and GL which stimulate the host cell's anabolic activity (e.g., by increased production of nucleotides and amino acids), thus providing additional metabolites to the intracellular pathogens, (c) switch to enhanced glutaminolysis and citrate lyase reaction reinforcing fatty acid/lipid biosynthesis, (d) modulations in lipid metabolism, including changes in the biosynthesis of steroid and eicosanoid hormones that alter signaling pathways subsequently leading to metabolic shifts. An overview on these host cell responses is also given in Figure 5.

As we repeatedly mentioned, the majority of the specific host responses are, however, only observed in *in vivo* infection models, but not in the frequently used established cell lines which represent cancer cells or artificially transformed cells. Most of these host cells have already experienced massive metabolic changes which may be similar to those triggered by the bacterial pathogens and hence may mask pathogen-induced host responses.

## Outlook

This review illustrates that we are still far away from an in-depth understanding how the host (cell) metabolism is changed during infection by bacterial pathogens and how these metabolic changes affect the outcome of the infection. In the last decade especially cancer research has produced a wealth of new insights into the metabolism of mammalian cells and its complex regulation (for recent reviews, see Smolková et al., 2011; Chen and Russo, 2012): (a) The involvement of certain protooncogenes and tumor suppressors, e.g., c-Myc and p53, as important transcriptional regulators of the genes encoding key enzymes for metabolic pathways and of miRNAs controlling their synthesis is now well-established (Cheung and Vousden, 2010; Dang, 2010; Gaglio et al., 2011). (b) Multiple specific hormone and nutrient receptors (“transceptors”) and signaling pathways have been shown to be involved in the regulation and modulation of cell metabolism and proliferation (Boulahbel et al., 2009; Dunlop and Tee, 2009; Hardie, 2011; Mosca et al., 2011).

Yet, not only these complex regulatory networks are of utmost importance for manipulating host cell metabolism, but also modulations in the activity of many enzymes involved in pathways of carbon-, nitrogen-, and energy metabolism. These modulations are often performed by phosphorylation, acetylation, or other post-translational reactions. In addition, many metabolic enzymes exist in different isoforms, and switches from one to another isoform may also alter metabolic fluxes.

It is likely, but not yet experimentally proven, that bacterial (especially intracellular) pathogens with their plethora of PAMPs and pathogenicity factors acting as effectors of host cell processes may influence the numerous regulatory host cell devices and/or the activity of metabolic enzymes by direct or indirect interaction. Indeed, some of the above described metabolic changes observed in host cells upon infection by the different bacterial pathogens could be readily explained by such interactions.

In-depth investigations on the interactions of metabolic host targets with components of the bacterial pathogens and the impact of these interactions on host cell metabolism certainly represent an exciting research area which may significantly extend our understanding of bacterial pathogenesis. Classic high throughput (“omics”) techniques will not suffice to solve these challenging problems. The adaptation and further development of new biochemical and physical techniques (in part described in this review) which allow the detection of cellular metabolic changes under *in vivo* conditions will be necessary for achieving this great task.

And last but not least—as repeatedly mentioned—it will be absolutely crucial to carry out these studies with appropriate *in vivo* models which make it possible to detect metabolic changes triggered by the bacterial pathogens during latent, acute, or chronic stages of infection.

### Conflict of interest statement

The authors declare that the research was conducted in the absence of any commercial or financial relationships that could be construed as a potential conflict of interest.
