# Supplementary material for: Metabolic host responses to infection by intracellular bacterial pathogens
Source: Front Cell Infect Microbiol. 2013 Jul 9;3:24. doi: 10.3389/fcimb.2013.00024 (PMC3705551; doi:10.3389/fcimb.2013.00024)
Supplement: Supplementary file 1 [file 54859__Presentation_1.PDF]

Supplements to

Metabolic host responses to infection by intracellular bacterial pathogens

Wolfgang Eisenreich, Jürgen Heesemann, Thomas Rudel and Werner Goebel

S1 *Catabolic pathways in eukaryotic cells*

Glucose is converted via glycolysis (GL), the pentose-phosphate pathway (PPP), and the tricarboxylic acid (TCA) cycle to metabolic intermediates, ATP and NAD(P)H (Fig. 2). The glycolytic reactions, catalyzing the oxidation of glucose to pyruvate, thereby generating ATP and NADH, take place in the cytosol of the eukaryotic cell. Under aerobic conditions pyruvate mainly enters the mitochondria and connects GL with the TCA cycle and the electron transport chain (ETC). These two pathways are localized to the mitochondrial matrix and the inner membrane, respectively. Pyruvate is first oxidized to acetyl-CoA by the pyruvate dehydrogenase complex (PDH). Acetyl-CoA is then channelled by citrate synthase (CS) into the TCA cycle where it is further oxidized to CO_2. The complete TCA cycle delivers 3 moles NADH, 1 mole $FADH_2$ and 1 mole GTP per mole acetyl-CoA, and generates several intermediates for biosynthetic reactions (Fig. 2). There are two forms of isocitrate-dehydrogenase (ICD), the mitochondrial ICD-1 uses NAD and the cytosolic ICD-2 employs NADP as electron acceptor. In eukaryotic cells, ICD-1 localizes to the mitochondrial matrix and participates in the TCA cycle, while the cytosolic ICD-2 generates NADPH for reductive anabolic reactions. NADH and $FADH_2$ are re-oxidized in the presence of oxygen via ETC where most of the free energy (ATP) from glucose is produced. Under limited oxygen ("hypoxic") conditions pyruvate is mainly converted by the NADH-dependent lactate dehydogenase (LDH) to lactate. The occurrence of isoforms is also observed for other catabolic enzymes, e.g. HK, PK, LDH, MDH and others (Fig. 2). Expression of the genes for a specific enzyme isoform may depend on the cell/tissue-type and/or on specific conditions in the given cell/tissue.

In most differentiated mammalian cells and tissues more than 80% of glucose is oxidized via GL and the remaining part via PPP, the alternative pathway for glucose oxidation. The latter pathway (Fig. 2) is independent of oxygen and does not generate ATP, but is essential for the production of NADPH. This coenzyme, required especially for anabolic reactions, is produced in the oxidative branch of the PPP which converts glucose-6P to 6-phosphogluconolactone and further to ribulose-5P. Ribose-5P generated from ribulose-5P by ribose-5P isomerase is needed for nucleotide biosynthesis. The non-oxidative branch of the PPP converts ribulose-5P into other phosphorylated C_3-, C_4-, C_5-, C_6-and C_7-sugars, two of which (fructose-6P and glyceraldehyde-3P) are also glycolytic intermediates which can be readily channelled into the GL. The cellular requirement for NADPH, ribose-5P and ATP determines the flow rates through GL and PPP.

PPP is also regulated by oxidative stress caused via ROS (reactive oxygen species, see below) which inactivate glyceraldehyde-3P dehydrogenase (Chuang et al., 2005), resulting in the redirection of the glucose flux from GL to the PPP. Increased glucose flux through PPP will lead to enhanced NADPH production, necessary for maintaining glutathione in its reduced form (GSH) which is required for the detoxification of increased ROS and hydrogen peroxide

concentrations, e.g. generated by hypoxia and infections (see below). The thereby generated glutathione disulfide (GSSG) must be regenerated to GSH by the NADPH-dependent glutathione reductase.

Glutamine may be used as alternative carbon source for maintenance of the TCA cycle. Glutaminolysis converts exogenous glutamine by glutaminase (GLS) to glutamate which is further oxidized in mitochondria by NAD-dependent glutamate dehydrogenase to α-KG. The latter intermediate is channeled into the TCA cycle generating additional TCA cycle intermediates and NADH for ATP production via the ETC (oxidative phosphorylation; OXPhOS). While most normal cells consume glutamine only to a small part via this anaplerotic pathway, it may represent a major catabolic pathway in many cancer cells (Wise et al., 2008).

In addition to glutamine, other glucogenic amino acids, as well as pyruvate, lactate and glycerol may also serve as carbon sources for energy and metabolite supply (via gluconeogenesis) in eukaryotic cells. All amino acids contain at least one nitrogen atom in the α-amino group and some contain additional nitrogen atoms in their side chains. Nitrogen cannot be used by mammalian cells in energy metabolism and the removal of nitrogen is typically the first step in amino acid degradation. This occurs by transamination which removes the α-amino group from the amino acid and transfers it to α-ketoglutarate (α-KG), leading to the accumulation of glutamate (Glu). The release of nitrogen from Glu may involve the urea cycle.

The transamination step leaves behind compounds that are different for each amino acid. Accordingly, each of the 20 amino acids has a more or less separate catabolic pathway. Most of these pathways result in the formation of pyruvate or intermediates of the TCA cycle [α-KG, succinyl-CoA (Suc), fumarate (Fum), oxaloacetate (Oxa) - see Fig. 2]. These intermediates can be either completely oxidized to CO_2 in the TCA cycle or used for glucose generation via gluconeogenesis (GN). The corresponding amino acids are therefore called glucogenic amino acids. Other degradation pathways produce acetoacetyl-CoA or acetyl-CoA that can enter the fatty acid and lipid biosynthesis. Phe, Tyr, Trp, Lys and Leu belong to these ketogenic amino acids, but Lys and Leu are the only amino acids that are solely ketogenic, giving rise only to acetyl-CoA or acetoacetyl-CoA and hence do not lead to net glucose production. Degradation of amino acids occurs in part in the cytosol and in part in the mitochondria.

Fatty acid degradation takes place exclusively in the mitochondria (Fig. 2). The major part of fatty acids is transferred from the cytosol via the acylcarnitine/carnitine carrier system to the matrix of mitochondria. Even-chain fatty acids are first oxidized via ß-oxidation to acetyl-CoA which can be completely oxidized to CO_2 in the TCA cycle. The electrons of the thereby produced $FADH_2$ and NADH are channeled into the respiration chain in the mitochondrial membrane generating large amounts of energy, e.g. oxidation of 1 molecule of palmitoyl-CoA generates more than 100 ATP. Complete oxidation of unsaturated fatty acids requires, in addition to the ß-oxidation enzymes, enoyl-CoA isomerase and eventually 2,4-dienoyl-CoA reductase. Oxidation of odd-chain fatty acids will always lead in addition to acetyl-CoA to the generation of propionyl-CoA which is finally converted via methylmalonyl-CoA to succinyl-CoA an intermediate of the TCA cycle. Catabolism of fatty acids (especially very long chain and branched chain fatty acids), can also occur in peroxisomes, organelles budding off the endoplasmic reticulum. The enzymatic reactions leading to acetyl-CoA are carried out by similar, but not identical enzymes, including a FAD-dependent dehydrogenase catalyzing the first degradation step; the electrons of the thereby generated $FADH_2$ are directly transferred to O_2 leading to H_2O_2 that is detoxified by catalase, an important peroxisomal enzyme, to H_2O and O_2. Acetyl-CoA and NADH produced in peroxisomes by fatty acid degradation are exported probably to mitochondria. Peroxisomes are also important organelles for the removal of excess purines as well as of polyamines and D-amino acids.

In the presence of excess of nutrients, glucose and fatty acids can be stored as glycogen and triglycerides (triacylglycerol, TAG), respectively. Synthesis of glycogen occurs mainly in muscle and liver cells by glycogen synthase (GYS1 and GYS2). Glycogen degradation (during carbon starvation conditions) is catalyzed by glycogen phosphorylase (PYG) whose activity is controlled, mainly by a cAMP-dependent phosphorylation cascade. TAG is formed in adipocytes and liver cells from diacylglycerol and acyl-CoA, catalyzed by the diacylglycerol O-acyltransferase (DGAT). This reaction is the terminal and the only regulated step in TAG synthesis and is responsible for the accumulation of adipose tissue. TAG yields more than twice as much energy for the same mass as do carbohydrates or proteins. When the organism needs fatty acids as energy source, breakdown of TAG (lipolysis) is catalyzed by several (cAMP or hormone-regulated) glyceride lipases in adipocytes (Chaves et al., 2011) releasing free fatty acids. TAG cannot pass through cell membranes freely and special lipases (lipoprotein lipases), localized at the wall of blood vessels, are required to break down TAG into free fatty acids and glycerol. The fatty acids can then be taken up by different cells via diffusion and specific transporters (see S3).

***S2** Anabolic and anaplerotic pathways in mammalian cells*

Many eukaryotic (in particular mammalian) cells import most of the low molecular precursors, i.e. amino acids, monomeric carbohydrates, fatty acids and nucleotides, needed for the biosynthesis of proteins, polysaccharides, lipids and nucleic acids, respectively from the environment by a large number of membrane-bound transporters (see below). However, if necessary, these cells are also capable of synthesizing several (so-called nonessential) amino acids, fatty acids, purine and pyrimidine nucleotides as well as porphyrines via conserved pathways. Glucose and other carbohydrates can also be synthesized by gluconeogenesis when nutrition is supported by alternative carbon sources, like glucogenic amino acids, lactate and glycerol. The essential reactions for gluconeogenesis (from pyruvate via oxaloacetate to glucose), are - besides the reversible enzymatic steps of the glycolytic pathway - the reactions catalyzed by pyruvate carboxylase (PC), phosphoenolpyuvate carboxykinase (PCK), fructo-1,6-bisphosphatase (FBP), and glucose-6-phosphatase (GP) leading to OXA, PEP, fructose-6P and glucose, respectively (Fig. 2).

Metabolites deriving from GL, PPP and TCA serve as precursors for these biosynthetic pathways. For this goal, some of the TCA metabolites are transported under certain conditions (see below) from mitochondria into the cytosol where they are further used for anabolic reactions (biosynthesis of fatty acid, lipids, amino acids). Citrate is transported to the cytosol and converted by ATP-dependent citrate lyase (ACL) to oxaloacetate and acetyl-CoA and further used for amino acid and lipid synthesis, respectively. Alternatively, citrate can be converted by cytosolic citrate dehydrogenase to isocitrate and further to α-KG by the cytosolic NADP-dependent ICD-2. Malate can be shuttled into the cytosol where it is oxidized to pyruvate and CO_2 by the cytosolic NADP-dependent malic enzyme (ME) (Fig. 2). Withdrawal of the TCA intermediates for biosynthetic purposes may cause the breakdown of the TCA cycle, unless the cycle is replenished in particular by the end products of amino acid catabolic pathways or by specific refilling (“anaplerotic”) reactions. As already mentioned above, glutaminolysis is an important anaplerotic pathway for refilling the TCA cycle, especially in many cancer cells (Wise et al., 2008). Another major anaplerotic reaction is the generation of oxaloacetate catalyzed by the mitochondrial ATP-dependent pyruvate carboxylase (PC).

In contrast to the above mentioned anabolic pathways, those leading to hormones and bile acids are specific achievements of vertebrates. Synthesis of steroid hormones and bile acids

branches off from the common steroid (cholesterol) biosynthesis pathway, while synthesis of eicosanoid hormones branches off the arachidonic acid pathway. The biosynthesis of these compounds requires several cytochrome P450-dependent hydroxylation steps and the CYP genes encode numerous members of the P450 family. The two classes of hormones can participate - among others - in the regulation of metabolism, e.g. via cAMP control.

***S3** Nutrient transporters in mammalian cells*

All mammalian cells possess multiple SLC transporters for glucose (Zhao and Keating, 2007; Thorens and Mueckler, 2010), for neutral, cationic and anionic amino acids (Hyde et al., 2003; Bröer, 2008). Long chain fatty acids (Bonen et al., 2002; Schwenk et al., 2010) and micronutrients; especially Fe- and Mg-ions (Schaible and Kaufmann, 2004; Garrick and Garrick, 2009) may be also transported by members of the SLC transporter family.
Glucose, as major carbon source for most mammalian cells, is transported by up to six, Na^+-dependent glucose transporters SGLT1-6 (determined by *SLC5A* genes) and 14 members of the family of facilitative carbohydrate GLUT transporters (determined by *SLC2A* genes). The distribution of these transporters is tissue-specific, e.g. SGLT1 is the only SLGT transporter expressed in the small intestine where it is found mainly on the apical membrane of enterocytes. SGLT1 is also found on renal S3 cells, whereas SGLT2 is located predominantly on the apical membrane of renal S1 and S2 cells (Kanai et al., 1994). The SGLT-mediated glucose transport occurs against the glucose concentration gradient using the Na^+-electrochemical gradient provided by the Na^+/K^+ ATP pump.
The GLUT-mediated sugar transport on the other hand utilizes the diffusion gradient of glucose (or other carbohydrates). The major members of the GLUT family transporting glucose are GLUT1 to GLUT4 which differ in their cellular localization and substrate affinity. GLUT1 is the most common isoform, highly expressed by endothelial cells, erythrocytes, astrocytes and by many tumor cells (Vander Heiden et al., 2009). GLUT2 is the major glucose transporter in liver and kidney and also present on the basolateral side of the polarized enterocytes (i.e. opposite to SGLT1). GLUT3 occurs mainly in the brain (Simpson et al., 2008) and GLUT4 (the insulin-responsive glucose transporter) is found in heart, adipopse tissue and skeletal muscle. Similar to GLUT1, the glucose transport capacity of GLUT4 seems to be regulated by ATP (Mohan et al., 2010). GLUT5 is the major fructose transporter, predominantly expressed on the apical membrane of enterocytes. The HMIT transporter, specific for myo-inositol also belongs to the GLUT family (Uldry et al., 2001). The other members of the SGLT and GLUT families are functionally less well characterized.

Amino acids are transported by mammalian cells via distinct “systems” (Bröer, 2008) which differ in substrate specificity and transport mechanism. Since the intracellular amino acid concentration is often higher than that in the extracellular surroundings, most amino acid transporters couple transport activity mainly to the Na^+ gradient (generated by the Na^+/K^+ ATPase), but also to the H^+ gradient or to the transmembrane potential. A single amino acid can be transported by more than one system. For example: Ala is transported by the Na^+-dependent “A-system” comprising various transporter isoforms (determined by *SLC38A* genes), which transport most short chain, neutral amino acids (i.e. Gly, Ala, Ser, Cys, Thr, Met), but Ala can be also transported by the Na^+-dependent “ASC-system” (two isoforms determined by *SLC1A* genes), the Na^+-independent “asc*-system” (SLC7A) and by still other systems that transport neutral amino acids. The anionic amino acids (especially Glu and Asp) are mainly transported by the Na^+-independent EAAT1-5 transporters (determined by *SLC1A* genes). Cationic amino acids (Asn, Gln, Arg, His) are mainly transported by CAT1-4, determined by the *SLC7A* genes (for further details, see Hyde et al., 2003; Bröer, 2008). Cell-

specific glutamine transport appears to be accomplished by four major transporters, including SNAT1, SNAT3, ASCT2 (essential for glutamine transport of rapidly growing epithelial cells and tumour cells in culture) and SLC6A19 (reviewed by McGivan and Bungard, 2007).

Fatty acids may diffuse passively through biomembranes, but various fatty acid binding proteins (FABPs) facilitate fatty acid transport across the membranes of many cell types and are involved in the delivery of fatty acids to specific sites within cells. Inside the cell, cytoplasmic fatty acid-binding proteins (FABPc) are involved in diffusion of FA from the plasma membrane to intracellular sites, such as the mitochondrial outer membrane, for further conversion. Six members of the SLC27 family facilitate membrane transport of long chain fatty acids (Stahl, 2004). Four *SLC* gene families are involved in mitochondrial transport, the most important one being the *SLC25* gene family. The mitochondrial carriers (43 members) shuttle a variety of metabolites between the mitochondrial matrix and the cytosol (Palmieri, 2004).

Iron (in form of Fe^{2+}or Fe^{3+}) is the most essential micronutrient acting as cofactor for many enzymes of basic metabolic pathways including the respiratory chain, but catalyzes also the formation of reactive oxygen species (ROS) and reactive nitrogen intermediates (RNI) which are highly toxic to cells. Iron occurs therefore in the cell only in bound forms linked to transferrin (TF), lactoferrin (LF) and ferritin or heme-bound in hemoproteins. The predominant form of iron taken up in the intestine is $Fe(OH)_2^+$ which is reduced to Fe^{2+} by duodenal cytochrome b reductase (DCYTB) before uptake by enterocytes via the divalent-metal transporter-1 (DMT-1; also termed SLC11A2 or NRAMP2). Members of the *SLC39* gene family also support the influx of Fe^{2+} in addition to Zn^{2+}, Cu^{2+} and Mn^{2+}.

Transferrin-complexed iron (TF-Fe^{2+}) binds to the TF receptor (TFR); the entire TFR-TF-Fe^{2+} complex is then endocytosed and Fe^{2+} released into the cytosol via DMT-1 associated with the endosomal membrane. Excess Fe^{2+} is either stored inside cells as ferritin or exported from enterocytes by ferroportin for circulation in the body, thereby provided to hepatocytes, macrophages, pneumocytes, renal cells and brain cells (Garrick and Garrick, 2009).

***S4**. NADPH oxidase and the inducible nitric oxide synthase (NOS2 in mammalian cells)*

NADPH oxidase is a membrane-bound enzyme complex which in its active form contains several proteins including five PHOX subunits (the membrane-integrated gp91^{phox} and p22^{phox}, and the cytosolic p40^{phox}, p47^{phox}, p67^{phox} and the GTPase Rac2, translocated to the membrane complex upon cell stimulation (Sumimoto et al., 2005). NADPH oxidase catalyzes the initial reaction of ROS production leading from O_2 to superoxide (O_2^-) which is further converted to other ROS, including H_2O_2, produced by superoxide dismutase (SOD), and hydroxyl radicals ($^{\bullet}OH$) and hydroxylanions (OH^-), produced by the iron-catalyzed Haber-Weiss reaction. ROS can be also produced by the mitochondrial ETC when electrons, which are normally transferred to cytochrome C oxidase (COX)-bound O_2 yielding H_2O, escape the ETC prematurely (at complex I or III) and react with O_2 to form O_2^- which is further converted by mitochondrial SOD into H_2O_2. This is under normal physiological conditions a rare reaction (1 to 2%), but is induced by stimuli, such as TNF-α, Ras or hypoxic conditions (Novo and Parola, 2008).

Inducible nitric oxide synthetase (iNOS or NOS2) is a cytosolic enzyme that in its homodimeric form synthesizes nitric oxide (NO) by utilising oxygen and electrons from NADPH to oxidize arginine to NO and citrulline.

Several enzymes are known that inactivate ROS, e.g. catalases and enzymes that synthesize, oxidize and reduce glutathione and thioredoxin. RNI production can be silenced by the

induction of arginase which competes with iNOS for the cellular L-arginine pool, thus balancing the generation of the cell-toxic RNI (Fang, 2004; Das et al., 2010). Heme oxygenase-1 (HO-1), catalysing the degradation of heme to biliverdin with the concurrent release of CO and ferrous iron, is another major antioxidant enzyme involved in cellular defense against oxidative and nitrosative stresses (Was et al., 2010).

S5. *Other Nutrient Sensors*

In addition to the above described major sensing systems still other nutrient sensors (often termed "transceptors") interacting with glucose, amino acids and fatty acids, may regulate energy and nutrient homeostasis. Transceptors are defined as proteins that bind, but do not necessarily transport basic nutrients and subsequently undergo conformational changes thereby triggering downstream reactions (Forsberg and Ljungdahl, 2001). Malfunction of any of these processes may significantly change metabolism and cause pathologiocal effects. As components of bacterial pathogens might interfere with these nutrient sensors, some basic aspects of this extensively studied field (Rolland et al., 2001; Holsbeeks et al., 2004; Raybould, 2008) are briefly summarized in the following.

Glucose sensing is best studied in yeast (*Saccharomyces cerevisiae*) where it is long known that high glucose concentration represses genes involved in metabolism of alternative carbohydrates, gluconeogenesis and respiration (Forsberg and Ljungdahl, 2001; Rolland et al., 2001). In *S. cerevisiae* glucose sensing involves (a) the hexokinase (Hxk2)-dependent pathway (Herrero et al., 1998), (b) the Snf3 and Rgt2 transceptors (glucose transporter homologues without transport functions)-dependent signaling pathway (Özcan et al., 1996; Holsbeeks et al., 2004) and (c) the cAMP-PKA pathway which requires the receptor Grp1 for sensing of extracellular glucose (Kraakman et al., 1999). Mammalian systems contain different types of glucose-sensing cells, including enterocytes and neurons. The glucokinase-based glucose sensing mechanism, well studied in pancreatic ß-cells, may also occur in other cell types (Yang et al., 1999). Recent findings suggest that some SGLTs may also act as glucose-sensing receptors in mammalian cells (Gribble et al., 2003).

Amino acids (AA) modulate essential cellular processes such as the own transport and metabolism, protein synthesis, proteolysis and hormone secretion via several signaling pathways. AA sensing is achieved by (a) AA-regulated kinases acting as intracellular AA sensors (Kilberg et al., 2005), (b) adaptive regulation of AA transporters sensing external AA availability (Hyde et al., 2003) and (c) G-protein-coupled receptors (GPCRs) that represent AA transporter-like sensors ("transceptors") linked to general signaling pathways, such as the protein kinase A (PKA) pathway (Holsbeeks et al., 2004; Hyde et al., 2003). Two proton-assisted amino acid transporters (PAT1 and PAT4) belonging to the SLC 36 family (see above) may function as transceptors for intracellular amino acids interacting with mTORC1 (Heublein et al., 2010). A model has been proposed in which the activation of mTORC1 by amino acids involves the recruitment of PAT (acting as transceptor) and RAG GTPases, (which can interact with mTORC1), to RHEB-containing late endosomal/lysosomal compartments. The resulting RHEB-GTP could subsequently activate mTORC1 (Avruch et al., 2009; Goberdhan, 2010).

Bioactive lipids, deriving mainly from cholesterol and fatty acids (FA), bind to nuclear receptors and influence metabolic functions, e.g. synthesis of several CYP enzymes and ABC transporters (Chawla et al., 2001). In addition, G-protein coupled receptors (GPCRs) have been recently identified (FFAR1-3 and GRP120) that seem to play a significant role in

sensing of free FA in the gastrointestinal tract (reviewed by Miyauchi et al., 2010). FFAR1 and GPR120 are activated by medium- and long-chain FA and mediate the secretion of incretin hormones, such as the glucagon-like peptide 1 (GLP-1) which stimulates insulin secretion and inhibits glucagon secretion (Drucker and Nauck, 2006). FFAR1 is also expressed in pancreatic ß-cells and contributes to the glucose-stimulated insulin secretion. Short-chain FA (i.e. acetate, propionate, butyrate) bind to FFAR2 and FFAR3 and these activated GPCRs control the release of the appetite-reducing peptide YY (PYY) from the ileum and colon.

S6. *Regulation of metabolic pathways in mammalian cells*

The involvement of the major regulatory factors in metabolism is summarized in the following in more detail as these regulators could serve again as targets for components of bacterial pathogens, thereby influencing host cell metabolism.

a. Allosteric regulation of catabolic enzymes

On the enzyme level glucose metabolism is mainly regulated by the activity of hexokinase (HK), phosphofructokinase (PFK1), pyruvate kinase (PK) and pyruvate dehydrogenase (PDH). There are three HK isoforms (HK1-3). HK activity is allosterically inhibited by high concentration of its end product, glucose-6-phosphate (G6P). HK1 and especially HK2 can interact with mitochondria by binding to the adenine nucleotide translocator (ANT) on the mitochondrial outer membrane. This allows the utilization of ATP, generated in the mitochondria and exported by ANT, for glucose phosphorylation (Koopman et al., 2010). Mitochondria-bound HKs are also less sensitive to G6P inhibition. PKF1 activity is inhibited by lactate and activated by fructose-2,6-bisphosphate (F2,6BP). Production of F2,6BP from F6P is catalyzed by 6-phosphofructo-2-kinase (PFK2) which may act also as phosphatase generating F6P from F2,6BP (see Fig.2). PFK2, occurring in different tissue-specific isoforms (Okar et al., 2001) does not participate directly in glycolysis, but strongly activates the glycolytic flux through its reaction product F2,6BP. The kinase activity of PFK2 is inhibited by PEP (Manes and El-Maghrabi, 2005). Pyruvate kinase (PK), converting PEP to pyruvate, exists also in various tissue-specific isoforms and is activated by the upstream glycolytic intermediate fructose-1,6-bisphosphate (F1,6BP) (Fenton and Hutchinson, 2009). In addition, PFK1 and PK are activated by low ATP/ADP+AMP levels and inhibited by high ATP/ADP+AMP levels. Pyruvate is a key metabolite in glucose catabolism (Fig. 1) which can be converted into lactate by lactate dehyrogenase (LDHA) or into acetyl-CoA by the pyruvate dehydrogenase complex (PDH) in mitochondria. PDH is inactivated by phosphorylation via PDH kinase (PDK1), thereby inhibiting the efficiency of the TCA cycle and of aerobic respiration.

b. Transcription factors and sensors controlling metabolism

PI3K/AKT

AKT, also known as protein kinase B (PKB), is a serine/threonine-specific protein kinase. Activation of AKT occurs via the signalling pathway triggered by insulin-, IGF-, EGF-receptors. The activity of the IR, IGF-R and EGF-R tyrosine kinases is first stimulated by the association with the growth factor ligands. The activated receptors recruit PI3 kinase (PI3K) which catalyzes the synthesis of the lipid second messenger phosphatidylinositol (3,4,5)-triphosphate (PIP3) and recruits AKT. AKT is activated by PIP3 and inhibited by the

phosphatase and tensin homolog (PTEN). The activated AKT stimulates glycolytic activity: (a) by increasing the transcription of *GLUT1* and the membrane translocation of the GLUT1 transporter, (b) by enhancing the activity of PFK2 (and hence indirectly PFK1 via F2,6BP) and (c) by stimulating HKII activity through mediating its association with the outer mitochondrial membrane (Barthel et al., 1999; Deprez et al., 1997). AKT also stimulates lipogenesis by enhancing ATP-dependent citrate lyase activity, a key enzyme of lipogenesis, generating acetyl-CoA from citrate (see above). Activated AKT suppresses fatty acid ß-oxidation by inducing the transcription and nuclear translocation of the transcriptional regulator SREBP (sterol regulatory element binding protein). Furthermore, PI3K/AKT through its interaction with the mammalian target of rapamycin (mTOR) is involved in cellular nutrient sensing (see below).

LKB1/AMPK and CREB/CRTC2

AMP-activated protein kinase (AMPK) is a cellular energy sensor which is activated by increasing cellular AMP/ATP ratio (indicating low energy). AMP allosterically alters AMPK conformation which triggers phosphorylation by LKB1 (or STK11), the constitutively active liver (serine/threonine) kinase B1 and known as tumour suppressor (Shackelford and Shaw, 2009). Phosphorylated AMPK switches on energy-delivering catabolic processes (glucose uptake and glycolysis, fatty acid oxidation) and switches off ATP consuming processes (protein synthesis, fatty acid and cholesterol biosynthesis). Activated AMPK also suppresses the mTOR complex 1 (Hardie, 2011).
The cAMP responsive factor CREB and its coactivator CRTC2 (initially called TORC2 - transducer of regulated CREB activity 2) plays also a central role in glucose homeostasis by regulating gluconeogenesis (for recent review, see Jitrapakdee, 2012). CRTC2 activity is directly linked to LBK1/AMPK. Unphosphorylated CRTC2 enters the nucleus and induces together with CREB the transcription of the genes essential for gluconeogenesis (Koo et al., 2005). On the other hand, CRTC2 is phosphorylated by activated AMPK which results in the sequestration of CRTC2 in the cytosol thereby blocking gluconeogenesis. Glucagon signaling promotes dephosphorylation of CRTC2 which induces transcription of the gluconeogenic genes in response to cAMP (Cheng and Saltiel, 2006).

The mammalian target of rapamycin (mTOR)

The mTOR signaling pathway is the most extensively studied intracellular target for sensing nutrient (especially amino acid) levels, stress and energy state and is thus an important regulator of metabolism and cell growth (reviewed by Wullschleger et al., 2006; Dunlop and Tee, 2009). Additional nutrient sensors exist in mammalian cells which are described in *S5*). The phylogenetically highly conserved mTOR protein, a large serine/threonine kinase of the PI3K family (Schmelzle and Hall, 2000; Avruch et al., 2006), exists in mammalians in two distinct complexes, mTORC1 and mTORC2. Both contain in addition to the common mTOR protein different proteins and exhibit different sensitivity towards rapamycin. The rapamycin sensitive mTORC1 contains as major components - in addition to the mTOR protein - the substrate binding RAPTOR (regulatory associated protein of TOR) and LST8 (lethal with sec13 protein 8, also termed GßL), PRAS40 (proline-rich AKT substrate of 40 kDa) and Deptor (DEP-domain-containing mTOR-interacting protein) (Laplante and Sabatini, 2009 ; Dunlop et al., 2009). The mTORC1 complex integrates via PI3K/AKT growth factor-signaling and via AMPK/LKB1 energy- and nutrient (especially amino acids)- response signals; mTORC1 thus controls metabolism and growth of mammalian cells by phosphorylation of several downstream substrates (Dunlop et al., 2009). The major mTORC1substrates are the p70 S6 kinases (S6K1 and S6K2) and the initiation factor 4E-

binding proteins (4E-BP1,2 and 3). The rapamycin-insensitive mTORC2 (not further discussed here) is also activated by growth factors, but not by nutrients.
The main positive regulator of mTORC1 is the GTP-charged form of RHEB, a RAS-like small GTPase. The Tuberous Sclerosis Complex (TSC), consisting of the heterodimer TSC1/TSC2, acts as GTPase and inhibits in its activated form RHEB-mediated mTORC1 signaling. Several signal cascades converge on TSC which thus acts as central coordinator of mTORC1 signaling activity (Dunlop et al., 2009). Insulin and insulin-like growth factors (IGF) modulate TORC1 activity by controlling GTP charging of RHEB by AKT-mediated TSC2 phosphorylation, whereas the energy sensing AMPK inhibits mTORC1 signal transduction by phosphorylation and activation of TSC2 (Corradetti et al., 2004). Several nutrient sensing pathways (see below) may either activate or inactivate mTORC1 signaling. Essential amino acids (in particular leucine) seem to regulate mTORC1 activity independent of AKT. Furthermore, HIF-1- dependent pathways inhibit mTORC1 under hypoxic conditions (Wouters and Koritzinsky, 2008).

The hypoxia inducible factor 1 (HIF-1)

HIF-1 is the central cellular oxygen sensor. Oxygen is indispensable for most eukaryotic multicellular organisms and fluctuations in oxygen levels will pose stress and may even threaten their viability. However, these organisms have evolved mechanisms which sense changes in oxygen levels and adapt the cell metabolism to the available oxygen supply. Under oxygen-limiting (“hypoxic”) conditions oxidative phosphorylation is reduced by inhibiting enzymes that fuel the TCA cycle (e.g. pyruvate dehydrogenase) and by stimulating glycolysis as a major pathway for ATP production through transcriptional up-regulation of genes encoding glycolytic enzymes and glucose transporters.
The cellular response to hypoxic conditions on the molecular level is mediated mainly by HIF-1, a heterodimeric transcription factor consisting of the constitutively expressed HIF-1*ß* (or ARNT) subunit and the oxygen-regulated HIF-1α subunit (Wang et al., 1995). Under normoxic conditions HIF-1α is hydroxylated on proline residues in its oxygen-dependent degradation domain by a prolyl hydroxylase domain-containing protein (PHD) which promotes ubiquitination and subsequent proteasomal degradation of HIF-1α. Under hypoxic conditions PHD-mediated hydroxylation of the HIF-1α subunit does not occur, resulting in HIF-1α stabilization and HIF-1α/HIF-1*ß* (ARNT) heterodimer formation (Hirsiliä et al., 2003; for details, see e.g. Nakayama, 2009). Recently, Boulahbel and coworkers (Boulahbel et al., 2009) provided evidence that PHDs may not only be an important oxygen-sensing system, but also act as signal transducers between amino acids and mTORC1 in animal cells. Thus, PHDs may function as metabolic sensors signaling not only to HIF-1, but also to mTORC1.
The hypoxia-stabilized HIF-1 binds (together with several other cofactors) to specific sites (hypoxia response elements, HRE) in the upstream region of the target genes and activate their transcription. Identified HIF-1 target genes (Patiar and Harris, 2006; Chi et al., 2006) include genes involved in glucose transport and catabolism (*GLUT1* and *3*; *HKI, HK2, GADH, PGK1, ALDO-A, ENO1, LDH-A*). Most of these genes are commonly regulated by HIF-1 and MYC (see below). But in contrast to MYC, HIF-1 inhibits indirectly also pyruvate dehydrogenase (PDH) by activating PDH kinase (PDK1) which phosphorylates and thereby inhibits PDH. Subsequently, formation of acetyl-CoA and its entry into the TCA cycle is impaired (Kim et al., 2006) resulting in reduced *de novo* fatty acid synthesis (Lum et al., 2007). HIF-1 also enhances electron transport chain efficiency by replacing COX4-2 for COX4-1 in the cytochrome c oxidase (COX) which results in increased ATP production and decreased ROS generation under hypoxic conditions (Fukuda et al., 2007). Other metabolic

genes activated by HIF-1 include the genes for transferrin (*TF*) and transferrin receptor (*TFRC*) involved in iron metabolism (Chi et al., 2006).

The oncogene MYC as regulator of metabolism

The protooncogene c-Myc (MYC) is a pleiotropic transcription factor that regulates many functions in mammalian cells, including metabolism (reviewed by Gordan et al., 2007). MYC is induced in normal cells by growth factors and is overexpressed in up to 70% of human tumour cells. Activated MYC upregulates transcription of *GLUT1*, glycolytic genes and *LDH-A* (Osthus et al., 2000). Direct binding of MYC to the conserved sites (E-boxes) has been demonstrated in the promoter upstream regions of the genes *HK*, *ENO* and *LDH-A* (Kim et al., 2004). MYC also induces a transcriptional program that promotes glutaminolysis by increasing the level of glutamine transporter and mitochondrial glutaminase (GLS) (Wise et al., 2008), leading to cell adaptation to glutamine as a bioenergetic substrate. MYC regulates the synthesis of several micro RNAs (miRNAs) on the transcriptional and posttranscriptional level, including miRNAs (especially miR-23a and miR-23b) that are involved in glucose and glutamine catabolism (Gao et al., 2009; Bui and Mendell, 2010).This corresponds well with the high consumption of glucose and glutamine in cancer cells with permanently active MYC. In this context, it is also of interest to note that the NF-kB p65 transcription factor may lead to a similar decreased expression of miR-23a (this miRNA represses the translation of the mRNA for GLS) and to concomitantly enhanced GLS synthesis (Rathore et al., 2012).

The expression of genes for key enzymes of several anabolic pathways, including nucleotide, fatty acid, polyamine and proline synthesis is also promoted by MYC either directly or via MYC-regulated miRNAs (Liu et al., 2008; Liu et al., 2012).

The tumour suppressor p53 as regulator of metabolism

The tumour repressor p53, in addition to its central function in cellular stress responses, is also an important regulator of metabolic pathways (Puzio-Kuter, 2011; Maddocks and Vousden, 2011). A key regulator of p53 stability is HDM2 which acts as an E3 ligase ubiquitinating p53 and thereby promoting its degradation (Patel and Player, 2008; Lee and Gu, 2010).

P53 plays a major role in glucose and (more indirectly by controlling cellular NADPH production) in lipid metabolism. As transcription factor, p53 inhibits glycolysis by down-regulation of *GLUT1* (and *GLUT4*) gene expression (Vousden and Ryan, 2009) and up-regulation of the *TIGAR* (tumor p53-induced glycolysis and apoptosis regulator) gene (Bensaad et al., 2006). TIGAR dephosphorylates fructose-1,6-bisphosphate (F1,6BP) and F2,6BP. Furthermore, p53 down-regulates the activity of 3-phosphoglycerate mutase (PGM) (Kondoh et al., 2005). These p53-mediated reactions inhibit the glycolytic flux. Recently, it was shown (Jiang et al., 2011) that cytosolic p53 protein can directly bind to glucose-6-phosphate dehydrogenase, thereby reducing the enzymatic activity of this key enzyme of the oxidative branch of the PPP (thus impairing NADPH production). On the other hand, p53 seems to favour the use of TCA for energy production and activates mitochondrial respiration by increasing the expression of *SCO2* (encoding cytochrome c oxidase) which enhances ATP production by aerobic respiration rather than by glycolysis (Matoba et al., 2006). In addition, p53 may stimulate mitochondrial fatty acid ß-oxidation and glutaminolysis under appropriate

conditions (Hu et al., 2010). In this context, it is noticeable that the p53 gene is the most frequently mutated tumour suppressor gene and loss of the p53 wild-type function explains many of the metabolic peculiarities of these tumour cells, including high glucose consumption, enhanced glycolysis, increased diversion of glucose into the oxidative branch of PPP, increased lipid metabolism and decreased mitochondrial respiration.

The role of autophagy in metabolism

Autophagy is a highly conserved and tightly regulated catabolic process which provides basic metabolites and maintains metabolic homeostasis in cells by degrading long-lived or damaged proteins and organelles (Klionsky, 2007; Rabinowitz and White, 2010). Autophagy is induced by a variety of extra- and intracellular stress stimuli, including nutrient starvation. This dynamic process includes membrane formation and fusion, leading to autophagosome formation, autophagosome-lysosome fusion, and subsequently to breakdown of the autophagosomal contents (which contains many cytosolic components) by lysosomal hydrolases. Thus, autophagy is in principle a protective mechanism that sustains cell survival under adverse conditions. Besides maintaining nutrient homeostasis, it is associated with other important cellular processes such as antigen presentation, defence against intracellular pathogens and longevity, but also with diseases (e.g. tumorigenesis, neurodegenerative diseases, cardiomyopathy, Crohn's disease, fatty liver, type II diabetes). Here, we consider only the "classical" autophagy process which is characterized by a non-selective degradation of the autophagosomal contents (for further information see recent reviews by Rouschop and Wouters, 2009; Tanida, 2010). The products of multiple autophagy-related genes (*ATG*) participate in this non-specific macroautophagy process (reviewed by Yang and Klionsky, 2009).
The regulation of autophagy is complex and not yet fully understood. Important regulators of autophagy are mTORC1 and HIF-1 (Nobukuni et al., 2007; Rabinowitz and White, 2010). Inhibition of mTORC1 by rapamycin and other inhibitory conditions (e.g. inhibition of glucose and amino acid uptake, low cellular energy status, increased ROS levels) activates autophagy. Autophagy is also induced under hypoxic conditions through a HIF-1 dependent mechanism (Mazure and Pouysségur, 2010) which requires expression of the HIF-1 dependent BNIP3 gene encoding the pro-apoptotic BH3-only proteins BNIP3 and BNIP3L. These two proteins displace Beclin1 from pro-apoptotic BCL-X_L and BCL-2 proteins which leads to autophagy. Beclin-1 (also known as autophagy-related protein ATG6) is required for the initiation of autophagosome formation. Under normoxic conditions, Beclin1 forms low-affinity complexes with BCL-X_L and BCL-2 via its BH3 domain, thereby decreasing the rate of autophagy. The affinity of the BH3 domains of the two Bnip proteins is too low to form tight complexes with BCL-X_L and BCL-2 and therefore fail to induce cell death. Recently, a link between glutaminolysis and regulation of autophagy has been established by showing that ammonia produced by glutaminase-catalyzed deamination of glutamine in mitochondria functions as stimulator of autophagic flux (Eng et al., 2010).

References to Supplements

Avruch J., Hara K., Lin, Y., Liu, M., Long, X., Ortiz-Vega, S., and Yonezawa, K. (2006). Insulin and amino-acid regulation of mTOR signaling and kinase activity through the Rheb GTPase. *Oncogene*. 25, 6361-72.

Avruch, J., Long, X., Ortiz-Vega, S., Rapley, J., Papageorgiou, A., and Dai, N. (2009). Amino acid regulation of TOR complex 1. *Am. J. Physiol. Endocrinol. Metab*. 296, E592-602.

Barthel, A., Okino, S.T., Liao, J., Nakatani, K., Li, J., Whitlock, J. P., and Roth, R. A. (1999). Regulation of GLUT1 gene transcription by the serine/threonine kinase Akt1. *J. Biol. Chem.* 274, 20281-6.

Bensaad, K., Tsuruta, A., Selak, M. A., Vidal, M. N., Nakano, K., Bartrons, R., Gottlieb, E., and Vousden, K. H. (2006). TIGAR, a p53-inducible regulator of glycolysis and apoptosis. *Cell* 126,107-20.

Bonen, A., Luiken, J. J., and Glatz, J. F. (2002). Regulation of fatty acid transport and membrane transporters in health and disease. *Mol. Cell. Biochem*. 239,181-92.

Boulahbel, H., Durán, R. V., and Gottlieb, E. (2009). Prolyl hydroxylases as regulators of cell metabolism. *Biochem. Soc. Trans.* 37, 291-4.

Bröer, S. (2008). Amino acid transport across mammalian intestinal and renal epithelia. *Physiol. Rev*. 88, 249-86.

Bui, T. V., and Mendell, J. T. (2010). Myc: Maestro of MicroRNAs. *Genes Cancer*. 1, 568-575.

Chaves, V. E., Frasson, D., and Kawashita, N. H. (2011). Several agents and pathways regulate lipolysis in adipocytes. *Biochimie.* 93, 1631-40.

Chawla. A., Repa, J. J., Evans, R. M., and Mangelsdorf, D. J. (2001). Nuclear receptors and lipid physiology: opening the X-files. *Science.* 294, 1866-70.

Cheng, A., and Saltiel, A. R. (2006). More TORC for the gluconeogenic engine. *Bioessays.* 28, 231-4.

Chuang, D. M., Hough, C., and Senatorov, V. V. (2005). Glyceraldehyde-3-phosphate dehydrogenase, apoptosis, and neurodegenerative diseases. *Annu. Rev. Pharmacol. Toxicol*. 45, 269-90.

Chi, J. T., Wang, Z., Nuyten, D. S., Rodriguez, E. H., Schaner, M. E., Salim, A., Wang, Y., Kristensen, G. B., Helland, A., Børresen-Dale, A. L., Giaccia, A., Longaker, M. T., Hastie, T., Yang, G. P., van de Vijver, M. J., and Brown P. O. (2006). Gene expression programs in response to hypoxia: cell type specificity and prognostic significance in human cancers. *PLoS Med.* 3, e47.

Corradetti, M. N., Inoki, K., Bardeesy, N., DePinho, R. A., and Guan, K. L. (2004). Regulation of the TSC pathway by LKB1: evidence of a molecular link between tuberous sclerosis complex and Peutz-Jeghers syndrome. *Genes Dev.* 18, 1533-8.

Das, P., Lahiri, A., Lahiri, A., Sen, M., Iyer, N., Kapoor, N., Balaji, K. N., and Chakravortty D. (2010). Cationic amino acid transporters and Salmonella Typhimurium ArgT collectively regulate arginine availability towards intracellular Salmonella growth. *PLoS One*. 5, e15466.

Deprez, J., Vertommen, D., Alessi, D. R., Hue, L., and Rider, M. H. (1997). Phosphorylation and activation of heart 6-phosphofructo-2-kinase by protein kinase B and other protein kinases of the insulin signaling cascades. *J. Biol. Chem.* 272, 17269-75.

Drucker, D. J., and Nauck, M. A. (2006). The incretin system: glucagon-like peptide-1 receptor agonists and dipeptidyl peptidase-4 inhibitors in type 2 diabetes. *Lancet*. 368, 1696-705.

Dunlop, E. A., Dodd, K. M., Seymour, L. A., and Tee, A. R. (2009). Mammalian target of rapamycin complex 1-mediated phosphorylation of eukaryotic initiation factor 4E-binding protein 1 requires multiple protein-protein interactions for substrate recognition. *Cell Signal.* 21, 1073-84.

Dunlop, E. A., and Tee, A. R. (2009). Mammalian target of rapamycin complex 1: signaling inputs, substrates and feedback mechanisms. *Cell Signal.* 21, 827-35.

Eng, C. H., Yu, K., Lucas, J., White, E., Abraham, R. T. (2010). Ammonia derived from glutaminolysis is a diffusible regulator of autophagy. *Sci. Signal*. 3, ra31.

Fang, F. C. (2004). Antimicrobial reactive oxygen and nitrogen species: concepts and controversies. *Nat. Rev. Microbiol*. 2, 820-32.

Fenton, A. W., and Hutchinson, M. (2009). The pH dependence of the allosteric response of human liver pyruvate kinase to fructose-1,6-bisphosphate, ATP, and alanine. *Arch. Biochem. Biophys.* 484, 16-23.

Forsberg, H., and Ljungdahl, P.O. (2001) Sensors of extracellular nutrients in Saccharomyces cerevisiae. *Curr Genet.* 40,91-109

Fukuda, R., Zhang, H., Kim, J. W., Shimoda, L., Dang, C. V., and Semenza, G. L. (2007). HIF-1 regulates cytochrome oxidase subunits to optimize efficiency of respiration in hypoxic cells. *Cell.* 129, 111-22.

Gao, P., Tchernyshyov, I., Chang, T. C., Lee, Y. S., Kita, K., Ochi, T., Zeller, K. I., De Marzo, A. M., Van Eyk, J. E., Mendell, J. T., and Dang, C. V. (2009). c-Myc suppression of miR-23a/b enhances mitochondrial glutaminase expression and glutamine metabolism. *Nature.* 458, 762-5.

Garrick, M. D., and Garrick, L. M. (2009). Cellular iron transport. *Biochim. Biophys. Acta*. 1790, 309-25.

Goberdhan, D. C. (2010). Intracellular amino acid sensing and mTORC1-regulated growth: new ways to block an old target? *Curr. Opin. Investig. Drugs*. 11, 1360-7.

Gordan, J. D., Thompson, C. B., and Simon, M. C. (2007). HIF and c-Myc: sibling rivals for control of cancer cell metabolism and proliferation. *Cancer Cell.* 12,108-13.

Gribble, F. M., Williams, L., Simpson, A. K., and Reimann, F. (2003). A novel glucose-sensing mechanism contributing to glucagon-like peptide-1 secretion from the GLUTag cell line. *Diabetes*. 52, 1147-54.

Hardie, D. G. (2011). Sensing of energy and nutrients by AMP-activated protein kinase. *Am. J. Clin. Nutr*. 93, 891S-6.

Herrero, P., Martínez-Campa, C., and Moreno, F. (1998). The hexokinase 2 protein participates in regulatory DNA-protein complexes necessary for glucose repression of the SUC2 gene in *Saccharomyces cerevisiae*. *FEBS* 434, 71-6.

Heublein, S., Kazi, S., Ogmundsdóttir, M. H., Attwood, E. V., Kala, S., Boyd, C. A., Wilson, C., and Goberdhan, D. C. (2010). Proton-assisted amino-acid transporters are conserved regulators of proliferation and amino-acid-dependent mTORC1 activation. *Oncogene.* 29, 4068-79.

Hirsilä, M., Koivunen, P., Günzler, V., Kivirikko, K. I., and Myllyharju, J. (2003). Characterization of the human prolyl 4-hydroxylases that modify the hypoxia-inducible factor. *J. Biol. Chem.* 278, 30772-80.

Holsbeeks, I., Lagatie, O., Van Nuland, A., Van de Velde, S., and Thevelein, J. M. (2004). The eukaryotic plasma membrane as a nutrient-sensing device. *Trends Biochem. Sci*. 29, 556-64.

Hu, W., Zhang, C., Wu, R., Sun, Y., Levine, A., and Feng, Z. (2010). Glutaminase 2, a novel p53 target gene regulating energy metabolism and antioxidant function. *Proc. Natl. Acad. Sci. U S A.* 107, 7455-60.

Hyde, R., Taylor, P. M., and Hundal, H. S. (2003). Amino acid transporters: roles in amino acid sensing and signaling in animal cells. *Biochem. J.* 373, 1-18.

Jiang, P., Du, W., Wang, X., Mancuso, A., Gao, X., Wu, M., and Yang, X. (2011). p53 regulates biosynthesis through direct inactivation of glucose-6-phosphate dehydrogenase. *Nat. Cell. Biol.* 13, 310-6.

Jitrapakdee, S. (2012). Transcription factors and coactivators controlling nutrient and hormonal regulation of hepatic gluconeogenesis. *Int. J. Biochem. Cell. Biol.* 44, 33-45.

Kanai, Y., Lee, W. S., You, G., Brown, D., and Hediger, M. A. (1994). The human kidney low affinity Na+/glucose cotransporter SGLT2. Delineation of the major renal reabsorptive mechanism for D-glucose. *J. Clin. Invest.* 93, 397-404.

Kilberg, M. S, Pan, Y. X., Chen, H., and Leung-Pineda, V. (2005). Nutritional control of gene expression: how mammalian cells respond to amino acid limitation. *Annu. Rev. Nutr*. 25, 59-85.

Kim, J. W., Tchernyshyov, I., Semenza, G. L., and Dang, C. V. (2006). HIF-1-mediated expression of pyruvate dehydrogenase kinase: a metabolic switch required for cellular adaptation to hypoxia. *Cell. Metab.* 3,177-85.

Kim, J. W., Zeller, K. I., Wang, Y., Jegga, A. G., Aronow, B. J., O'Donnell, K. A., and Dang, C. V. (2004). Evaluation of myc E-box phylogenetic footprints in glycolytic genes by chromatin immunoprecipitation assays. *Mol. Cell. Biol.* 24, 5923-36.

Klionsky, D. J. (2007). Autophagy: from phenomenology to molecular understanding in less than a decade. *Nat. Rev. Mol. Cell. Biol*. 8, 931-7.

Kondoh, H., Lleonart, M. E., Gil, J., Wang, J., Degan, P., Peters, G., Martinez, D., Carnero, A., and Beach, D. (2005). Glycolytic enzymes can modulate cellular life span. *Cancer Res.* 65, 177-85.

Koo, S. H., Flechner, L., Qi, L., Zhang, X., Screaton, R. A., Jeffries, S., Hedrick, S., Xu, W., Boussouar, F., Brindle, P., Takemori, H., Montminy, M. (2005). The CREB coactivator TORC2 is a key regulator of fasting glucose metabolism. *Nature.* 437, 1109-11.

Koopman, W. J., Nijtmans, L. G., Dieteren, C. E., Roestenberg, P., Valsecchi, F., Smeitink, J. A,, and Willems, P. H. (2010). Mammalian mitochondrial complex I: biogenesis, regulation, and reactive oxygen species generation. *Antioxid. Redox Signal.* 12, 1431-70.

Kraakman, L., Lemaire, K., Ma, P., Teunissen, A. W., Donaton, M. C., Van Dijck, P., Winderickx, J., de Winde, J. H., and Thevelein, J. M. (1999). A *Saccharomyces cerevisiae* G-protein coupled receptor, Gpr1, is specifically required for glucose activation of the cAMP pathway during the transition to growth on glucose. *Mol. Microbiol.* 32, 1002-12.

Laplante, M., and Sabatini, D. M. (2009). mTOR signaling at a glance. *J. Cell. Sci.* 122, 3589-94.

Lee, J. T., and Gu, W. (2010). The multiple levels of regulation by p53 ubiquitination. *Cell. Death Differ.* 17, 86-92.

Liu, Y. C., Li, F., Handler, J., Huang, C. R., Xiang, Y., Neretti, N., Sedivy, J. M., Zeller, K. I., and Dang, C. V. (2008). Global regulation of nucleotide biosynthetic genes by c-Myc. *PLoS One*. 3, e2722.

Liu, W., Le, A., Hancock, C., Lane, A. N., Dang, C. V,, Fan, T. W., and Phang, J. M. (2012). Reprogramming of proline and glutamine metabolism contributes to the proliferative and metabolic responses regulated by oncogenic transcription factor c-MYC. *Proc. Natl. Acad. Sci. U S A.* 109, 8983-8.

Lum, J. J,, Bui, T., Gruber, M., Gordan, J. D., DeBerardinis, R. J., Covello, K. L., Simon, M. C., and Thompson, C. B. (2007). The transcription factor HIF-1alpha plays a critical role in the growth factor-dependent regulation of both aerobic and anaerobic glycolysis. *Genes Dev.* 21, 1037-49.

Maddocks, O. D., and Vousden, K. H. (2011). Metabolic regulation by p53. *J. Mol. Med. (Berl).* 89, 237-45.

Manes, N. P., and El-Maghrabi, M. R. (2005). The kinase activity of human brain 6-phosphofructo-2-kinase/fructose-2,6-bisphosphatase is regulated via inhibition by phosphoenolpyruvate. *Arch. Biochem. Biophys*. 438, 125-36.

Matoba, S., Kang, J. G., Patino, W. D, Wragg, A., Boehm, M., Gavrilova, O., Hurley, P. J., Bunz, F., and Hwang, P. M. (2006). p53 regulates mitochondrial respiration. *Science.* 312, 1650-3.

Mazure, N. M., and Pouysségur J. (2010). Hypoxia-induced autophagy: cell death or cell survival? *Curr. Opin. Cell. Biol*. 22, 177-80.

McGivan, J. D., and Bungard, C. I. (2007). The transport of glutamine into mammalian cells. *Front. Biosci.* 12, 874-82.

Miyauchi, S., Hirasawa, A., Ichimura, A., Hara, T., and Tsujimoto, G. (2010). New frontiers in gut nutrient sensor research: free fatty acid sensing in the gastrointestinal tract. *J. Pharmacol. Sci.* 112, 19-24.

Mohan, S., Sheena, A., Poulose, N., and Anilkumar, G. (2010). Molecular dynamics simulation studies of GLUT4: substrate-free and substrate-induced dynamics and ATP-mediated glucose transport inhibition. *PLoS One.* 5, e14217.

Nakayama, K. (2009). Cellular signal transduction of the hypoxia response. *J. Biochem.* 146, 757-65.

Nobukuni, T., Kozma, S. C., and Thomas, G. (2007). hvps34, an ancient player, enters a growing game: mTOR Complex1/S6K1 signaling. *Curr. Opin. Cell. Biol.* 19, 135-41.

Novo, E, and Parola, M. (2008). Redox mechanisms in hepatic chronic wound healing and fibrogenesis. *Fibrogenesis Tissue Repair*. 1, 5.

Özcan, S., Dover, J., Rosenwald, A. G., Wölfl, S., and Johnston, M. (1996). Two glucose transporters in *Saccharomyces cerevisiae* are glucose sensors that generate a signal for induction of gene expression. *Proc. Natl. Acad. Sci. U S A.* 93, 12428-32.

Okar, D. A., Manzano, A., Navarro-Sabatè, A., Riera, L., Bartrons, R., and Lange, A. J. (2001). PFK-2/FBPase-2: maker and breaker of the essential biofactor fructose-2,6-bisphosphate. *Trends Biochem. Sci.* 26, 30-5.

Osthus, R. C., Shim, H., Kim, S., Li, Q., Reddy, R., Mukherjee, M., Xu, Y., Wonsey, D., Lee, L. A., and Dang, C. V. (2000). Deregulation of glucose transporter 1 and glycolytic gene expression by c-Myc. *J. Biol. Chem.* 275, 21797-800.

Palmieri, F. (2004). The mitochondrial transporter family (SLC25): physiological and pathological implications. *Pflügers Arch*. 447, 689-709.

Patel, S., and Player, M. R. (2008). Small-molecule inhibitors of the p53-HDM2 interaction for the treatment of cancer. *Expert Opin. Investig. Drugs.*17, 1865-82.

Patiar, S., and Harris, A. L. (2006). Role of hypoxia-inducible factor-1alpha as a cancer therapy target. *Endocr. Relat. Cancer.* 13 Suppl. 1, S61-75.

Puzio-Kuter, A. M. (2011). The role of p53 in metabolic regulation. *Genes Cancer.* 2, 385-91.

Rabinowitz, J. D, and White, E. (2010). Autophagy and metabolism. *Science.* 330, 1344-8.

Rathore, M. G., Saumet, A., Rossi, J. F., de Bettignies, C., Tempé, D., Lecellier, C. H., and Villalba, M. (2012). The NF-κB member p65 controls glutamine metabolism through miR-23a. *Int. J. Biochem. Cell. Biol.* 44, 1448-56.

Raybould, H. E. (2008). Nutrient sensing in the gastrointestinal tract: possible role for nutrient transporters. *J. Physiol. Biochem.* 64, 349-56.

Rolland, F., Winderickx, J., and Thevelein, J. M. (2001). Glucose-sensing mechanisms in eukaryotic cells. *Trends Biochem. Sci.* 26, 310-7.

Rouschop, K. M., and Wouters, B. G. (2009). Regulation of autophagy through multiple independent hypoxic signaling pathways. *Curr. Mol. Med.* 9, 417-24.

Schaible, U. E., and Kaufmann, S. H. (2004). Iron and microbial infection. *Nat. Rev. Microbiol*. 2, 946-53.

Schmelzle, T., and Hall, M. N. (2000). TOR, a central controller of cell growth. *Cell.* 103, 253-62.

Schwenk, R. W., Holloway, G. P., Luiken, J. J., Bonen, A., and Glatz, J. F. (2010). Fatty acid transport across the cell membrane: regulation by fatty acid transporters. *Prostaglandins Leukot. Essent. Fatty Acids*. 82, 149-5.

Shackelford, D. B., and Shaw, R. J. (2009). The LKB1-AMPK pathway: metabolism and growth control in tumour suppression. *Nat. Rev. Cancer.* 9, 563-75.

Simpson, I. A., Dwyer, D., Malide, D., Moley, K. H., Travis, A., and Vannucci, S. J. (2008). The facilitative glucose transporter GLUT3: 20 years of distinction. *Am. J. Physiol. Endocrinol. Metab.* 295, E242-53.

Stahl, A. (2004). A current review of fatty acid transport proteins (SLC27). *Pflügers Arch.* 447, 722-7.

Sumimoto, H., Miyano, K., and Takeya, R. (2005). Molecular composition and regulation of the Nox family NAD(P)H oxidases. *Biochem. Biophys. Res. Commun.* 338, 677-86.

Tanida, I. (2011). Autophagosome formation and molecular mechanism of autophagy. *Antioxid. Redox Signal*. 14, 2201-14.

Thorens, B., and Mueckler, M. (2010). Glucose transporters in the 21st Century. *Am. J. Physiol. Endocrinol. Metab.* 298, E141-5.

Uldry, M., Ibberson, M., Horisberger, J. D., Chatton, J. Y., Riederer, B. M, and Thorens, B. (2001). Identification of a mammalian H(+)-myo-inositol symporter expressed predominantly in the brain. *EMBO J.* 20, 4467-77.

Vander Heiden, M. G., Cantley, L. C., and Thompson, C. B. (2009). Understanding the Warburg effect: the metabolic requirements of cell proliferation. *Science.* 324, 1029-33.

Vousden, K. H., and Ryan, K. M. (2009). p53 and metabolism. *Nat. Rev. Cancer.* 9, 691-700.

Wang, G. L., Jiang, B. H., Rue, E. A., and Semenza, G. L. (1995). Hypoxia-inducible factor 1 is a basic-helix-loop-helix-PAS heterodimer regulated by cellular O_2 tension. *Proc. Natl. Acad. Sci. U S A.* 92, 5510-4.

Was, H., Dulak, J., and Jozkowicz, A. (2010). Heme oxygenase-1 in tumor biology and therapy. *Curr. Drug Targets.* 11, 1551-70.

Wise, D. R., DeBerardinis, R. J., Mancuso, A., Sayed, N., Zhang, X. Y., Pfeiffer, H. K., Nissim, I., Daikhin, E., Yudkoff, M., McMahon, S. B., and Thompson, C. B. (2008). Myc regulates a transcriptional program that stimulates mitochondrial glutaminolysis and leads to glutamine addiction. *Proc. Natl. Acad. Sci. U S A.*105, 18782-7.

Wouters, B. G., and Koritzinsky, M. (2008). Hypoxia signaling through mTOR and the unfolded protein response in cancer. *Nat. Rev. Cancer*. 8, 851-64.

Wullschleger, S., Loewith, R., and Hall, M. N. (2006). TOR signaling in growth and metabolism. *Cell.* 124, 471-84.

Yang, Z., and Klionsky, D. J. (2009). An overview of the molecular mechanism of autophagy. *Curr. Top. Microbiol. Immunol*. 335, 1-32.

Yang, X. J., Kow, L. M., Funabashi, T., and Mobbs, C. V. (1999). Hypothalamic glucose sensor: similarities to and differences from pancreatic beta-cell mechanisms. *Diabetes* 48, 1763–72.

Zhao, F. Q., and Keating, A. F. (2007). Functional properties and genomics of glucose transporters. *Curr. Genomics*. 8, 113-28.
